# Souffle/Spastizin Controls Secretory Vesicle Maturation during Zebrafish Oogenesis

**DOI:** 10.1371/journal.pgen.1004449

**Published:** 2014-06-26

**Authors:** Palsamy Kanagaraj, Amandine Gautier-Stein, Dietmar Riedel, Christoph Schomburg, Joan Cerdà, Nadine Vollack, Roland Dosch

**Affiliations:** 1 Institut fuer Entwicklungsbiochemie, Georg-August Universitaet Goettingen, Goettingen, Germany; 2 Departement de Zoologie et Biologie Animale, Universite de Geneve, Geneva, Switzerland; 3 Max-Planck Institut fuer Biophysikalische Chemie, Goettingen, Germany; 4 IRTA-Institute of Marine Sciences, CSIC, Barcelona, Spain; Hubrecht Institute, Netherlands

## Abstract

During oogenesis, the egg prepares for fertilization and early embryogenesis. As a consequence, vesicle transport is very active during vitellogenesis, and oocytes are an outstanding system to study regulators of membrane trafficking. Here, we combine zebrafish genetics and the oocyte model to identify the molecular lesion underlying the zebrafish *souffle* (*suf*) mutation. We demonstrate that *suf* encodes the homolog of the Hereditary Spastic Paraplegia (HSP) gene *SPASTIZIN* (*SPG15*). We show that in zebrafish oocytes *suf* mutants accumulate Rab11b-positive vesicles, but trafficking of recycling endosomes is not affected. Instead, we detect Suf/Spastizin on cortical granules, which undergo regulated secretion. We demonstrate genetically that Suf is essential for granule maturation into secretion competent dense-core vesicles describing a novel role for Suf in vesicle maturation. Interestingly, in *suf* mutants immature, secretory precursors accumulate, because they fail to pinch-off Clathrin-coated buds. Moreover, pharmacological inhibition of the abscission regulator Dynamin leads to an accumulation of immature secretory granules and mimics the *suf* phenotype. Our results identify a novel regulator of secretory vesicle formation in the zebrafish oocyte. In addition, we describe an uncharacterized cellular mechanism for Suf/Spastizin activity during secretion, which raises the possibility of novel therapeutic avenues for HSP research.

## Author Summary

Oocytes of egg laying animals frequently represent the biggest cell type of a species. The size of the egg is a consequence of active transport processes, *e.g.* the import of yolk proteins, which results in the massive storage of vesicles. In addition, secretory vesicles termed cortical granules are stored in the oocyte to be discharged right after fertilization during cortical reaction, which also occurs in mammals. Their secretion leads to chorion expansion, which prevents the lethal entry of additional sperm and protects the developing embryo against physical damage. Mutants with a defect in membrane transport are successful tools to discover genes regulating vesicle formation. We molecularly identify the disrupted gene in the recessive maternal-effect mutation *souffle*, which encodes a homolog of human SPASTIZIN. SPASTIZIN was previously implicated in endocytosis, but our cellular analysis of mutant oocytes connects this gene also with the regulation of cortical granule exocytosis. More precisely, we show that Suf/Spastizin is crucial for the maturation of cortical granules into secretion competent vesicles describing a novel role for this protein. Since SPASITIZN causes the disease Hereditary Spastic Paraplegia in humans, our results will help to decipher the pathogenesis of this neurodegenerative disorder.

## Introduction

Oogenesis prepares the egg to start the development of a new organism. During their development, oocytes actively import and secrete proteins using the basic cellular mechanism of vesicle transport [1]–[3]. As a consequence, oocytes contributed substantially to our understanding of vesicle trafficking *e.g.* Clathrin-coated vesicles were first described in mosquito oocytes [4]. Moreover, a plethora of novel regulators were discovered by exploiting the genetics of the *Caenorhabditis elegans* oocyte [5]–[9]. In zebrafish, oogenesis starts with a burst of secretory vesicle formation, which are called cortical granules [10], [11]. Shortly afterwards, oocytes endocytose enormous amounts of the yolk precursor protein Vitellogenin (Vtg) by receptor-mediated endocytosis increasing its volume about 3000-fold within ten days [12], [reviewed in 13]. Hence, the zebrafish oocyte provides the opportunity to integrate vertebrate genetics to visualize active trafficking of abundant and large vesicles in one big cell.

In humans, defects in vesicle trafficking lead to neurodegenerative diseases [14]–[16]. The disorder Hereditary Spastic Paraplegia (HSP) is characterized by progressive loss of lower limb motility [17]–[19]. At the cellular level, this spasticity is caused by the axonal degeneration of neurons in the corticospinal tracts, which are considered the longest axons in the human body. However, the cellular analysis of the dying neurons is hampered by the adult onset of the disease and the complexity of the nervous system. Therefore, the precise cellular mechanism for most HSP genes is currently controversial.

One of the HSP genes with multiple cellular roles is *SPG15*, which encodes SPASTIZIN aka ZFYVE26 or FYVE-CENT [20], [21]. In a cell culture RNAi screen, SPASTIZIN was shown to be necessary for cytokinesis, but this defect is not observed in human and murine mutants [21], [22]. This cell culture study found that the endogenous protein localizes to centrosomes and the midbody during cytokinesis of human fibroblasts. In neuronal tissue culture, SPASTIZIN was shown to localize to microtubules, ER, mitochondria and endosomal vesicles [22], [23]. In additional tissue culture reports, SPASTIZIN interacted with the UVRAG complex during DNA repair [24] and autophagosome maturation [25]. Furthermore, SPASTIZIN binds to the recently discovered adaptor protein complex 5 (AP5) regulating multivesicular body (MVB) formation [26], [27]. Although these localization data in different tissue culture cells are not mutually exclusive, it is difficult to reconcile the described cellular functions of SPASTIZIN in fibroblasts into an underlying cellular defect causing neurodegeneration in neuronal cells.

Mutants provide an essential tool to determine the endogenous role of a gene. Previously, we used a mutagenesis screen in zebrafish to discover vertebrate regulators of egg development and early embryogenesis, which isolated the *souffle* (*suf*) mutation named after its defect during oogenesis [28]. Here, we positionally cloned *suf* and show that it encodes the homolog of SPASTIZIN. In *suf* mutants, we show that oocytes expand a Rab11b-positive compartment, but correctly transport the recycling cargo Transferrin. Moreover, Suf colocalizes with Rab11b on secretory vesicles called cortical granules in the oocyte. We demonstrate genetically that Suf/Spastizin is essential for the formation of cortical granules. Importantly, our subcellular analysis indicates that loss of Suf/Spastizin inhibits vesicle maturation, probably during sorting, which is necessary to complete the formation of Clathrin-coated buds and eventually to pinch-off vesicles. Finally, blocking vesicle scission with the pharmacological Dynamin-inhibitor Dynasore mimics the mutant phenotype supporting the hypothesis that Suf is required for vesicle fission, which is critical for the maturation of secretory granules in the egg. Collectively, these results identify Suf/Spastizin as a novel key gene controlling the maturation of cortical granules in zebrafish oocytes, which may also bring us closer to understand the cellular etiology of HSP.

## Results

### Souffle encodes the zebrafish homolog of SPASTIZIN

**Figure 1:**
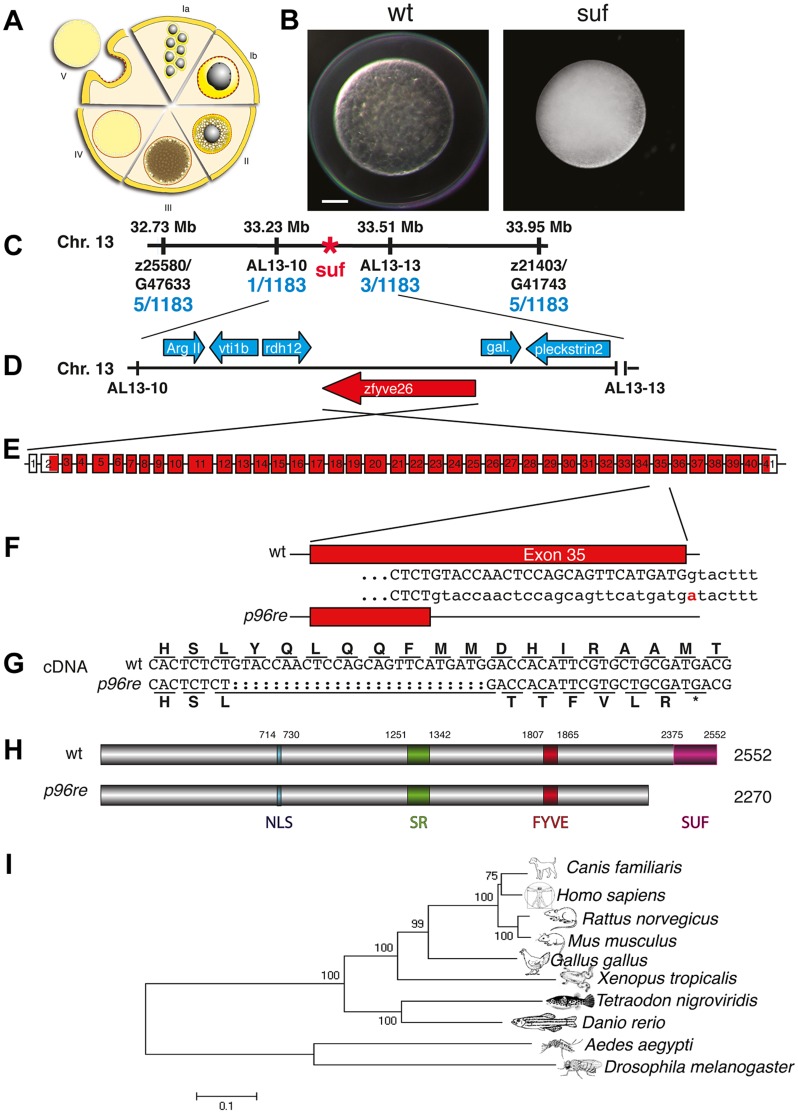
Molecular identification of the *suf* locus. (A) Diagram of zebrafish oogenesis represented as a composite of wedges displaying a single stage [11]. Oocytes are not drawn to scale and in the ovary, stages are mixed. Ia: 7–20 µm diameter of the oocyte; oocytes are still connected forming oogonia after the last mitosis. Ib: 20–140 µm, oocytes are separated by a layer of follicle cells (dashed red-yellow line) and several nucleoli appear in the nucleus (grey). II: 140–340 µm, formation of cortical granules is initiated (white vesicles). III: 340–690 µm, massive accumulation of yolk globules (brown vesicles) during vitellogenesis covering the germinal vesicle (oocyte nucleus). IV: 690–730 µm, oocytes are transformed into a fertilizable egg by meiotic maturation inducing germinal-vesicle-break-down. Simultaneously, the cytoplasm becomes transparent by yolk protein cleavage. V: 730–750 µm, eggs are released from the follicle layer of the ovary during ovulation into the oviduct. (B) Stage V oocytes 10 min after activation from heterozygous wild-type (wt; left panel) and mutant mothers (*suf*; right panel) showing the opaque cytoplasm and the chorion elevation defect in the mutant. Scale bar: 100 µm. (C) Genetic map of the *suf*-locus on chromosome 13. Meiotic mapping located the mutation between the markers z25580/G47633 (0.4 cM, centiMorgan; 5 recombinants/1183 females) and z21403/G41743 (0.4 cM) corresponding to a physical interval of 1.22 Mb (megabases). Fine mapping of the *suf*-locus identified markers AL13-10 (0.08 cM) and AL13-13 (0.2 cM), which physically represents 280 kB. (D) Among other genes, this interval contains the ArgII, vti1b, rdh12, galectin and pleckstrin2 genes, whose cDNA sequence did not display mutations in comparison to the database genome (http://www.ensembl.org/Danio_rerio/Info/Index). (E) Exon-intron structure of the *suf* gene. (F) The *p96re* allele carries a G-A transition at the genomic level destroying a splice donor site in the transcribed RNA. (G) The selection of an alternative, upstream splice donor leads to a deletion of 25 nucleotides causing a frameshift in the cDNA encoding six aberrant amino acids and eventually creating a premature STOP-codon (asterisk). (H) The mutant protein is predicted to lack 282 amino acids at the C-terminus including the conserved SUF domain from amino acid 2375 to 2552. (I) Phylogenetic diagram displaying the conservation of Souffle proteins among vertebrates. The *Anopheles* and *Drosophila* proteins were used to root the tree. Numbers indicate bootstrap-values and the scale the number of substitutions per amino acid residue.

Contrary to transparent eggs of wild-type (wt) mothers, maternal *suf* mutants spawn opaque eggs, which fail to proteolytically cleave yolk proteins [28]. In addition, mutant stage V eggs did not elevate their chorion after activation as observed in wt (Figure 1A, B). With this early defect the *suf* mutation rather causes a female-sterile than a maternal-effect phenotype. To characterize the molecular mechanism of the defect, we positionally cloned the disrupted gene in *suf* mutants. We previously located the *suf* mutation on chromosome 13 of the zebrafish genome. Genotyping 1183 females identified 5 recombinants with the SSLP marker z25580/G47633 and another 5 recombinants with z21403/G41743 restricting the critical interval with the *suf* mutation to 1.22 Mb (Figure 1C). Generating novel SSLP markers AL13-10 and AL13-13, we reduced the interval to 270 kb. Within this genomic region, we sequenced the cDNAs of *arginase*, *vti1b* (*t-snare*), *rdh12* (*retinol dehydrogenase* 12), *zfyve26* (*spastizin*), *galectin* and *pleckstrin2* (Figure 1D). The *zfyve26* gene consists of 41 exons and encodes a predicted mRNA of 7798 bp (Figure 1E). Comparing the *zfyve26* cDNA-sequence between wild type and the *p96re* allele of *suf* mutants showed a 25 bp deletion at the 3′-end of exon 35 (Figure 1G). However, the genomic sequence revealed a single point mutation in a splice donor, which probably results in cryptic splice donor selection 25 bp upstream of the wt splice site (Figure 1F). The deletion of 25 bp results in a frameshift, which creates a termination codon after six aberrant amino acids (Figure 1G). The premature STOP deletes 282 of the 2552 amino acids in the predicted Zfyve26 protein resulting in a shortened protein of 2270 amino acids (Figures 1H). Searching for protein motifs with the Prosite database [29] and MyHits [30] revealed a bipartite nuclear localization signal (amino acid 714–730), a serine-rich domain (aa 1251–1342) and a zinc finger FYVE domain (aa 1807–1865). FYVE domains bind to the lipid phospatidylinositol-3-phosphate (PI3P) predominantly present on endosomes [31]–[34]. Phylogenetic analysis detected one homolog in most metazoan genomes and a similar gene in *Drosophila melanogaster* (CG5270), albeit no homolog in the *C. elegans* genome (Figure 1I). Remarkably, although teleosts underwent an additional genome duplication compared to tetrapods [35], all ten teleost genomes available at the Ensembl-database (http://www.ensembl.org) contain a single paralog of Suf/Spastizin, which we confirmed for zebrafish by BLAST searches with full-length Suf (data not shown). Alignment of the vertebrate protein sequences discovered a highly conserved C-terminus, which we termed Suf-domain. Scanning the zebrafish genome with the Suf-domain did not detect other proteins with a similar amino acid motif. This motif is also conserved in plants, but the protein does not contain a FYVE-domain (data not shown). In human tissue culture cells, this domain in SPASTIZIN interacts with BECLIN1, KIF13A and TTC19 [21], [36] and is predicted to form alpha-helical solenoids as found in Clathrin heavy chain [27]. Since the Suf domain is deleted in the *p96re* allele, it is essential for Suf function during zebrafish oogenesis. Together, these data identified *souffle* as the zebrafish homolog of *SPASTIZIN* [20].

### *Souffle*/*spastizin* is maternally expressed

HSP patients require SPASTIZIN in the nervous system [20] and similarly, a recent mouse k.o. shows an adult onset neurodegeneration but not an embryological defect [22]. In zebrafish, inhibiting Spastizin by injection of morpholino-oligonucleotides (MO) leads to embryogenesis defects such as a twisted tails [37]. A fraction of these embryos without morphological changes show motor axon outgrowth failure leading to reduced motility, but are not paralyzed. Unexpectedly, we did not detect a neurological phenotype in zygotic zebrafish *suf* −/− embryos such as a touch response defect or a failure in motor axon outgrowth (data not shown). However, upon MO-injection, we confirmed the previously reported severe phenotypes (Figure S1).

**Figure 2:**
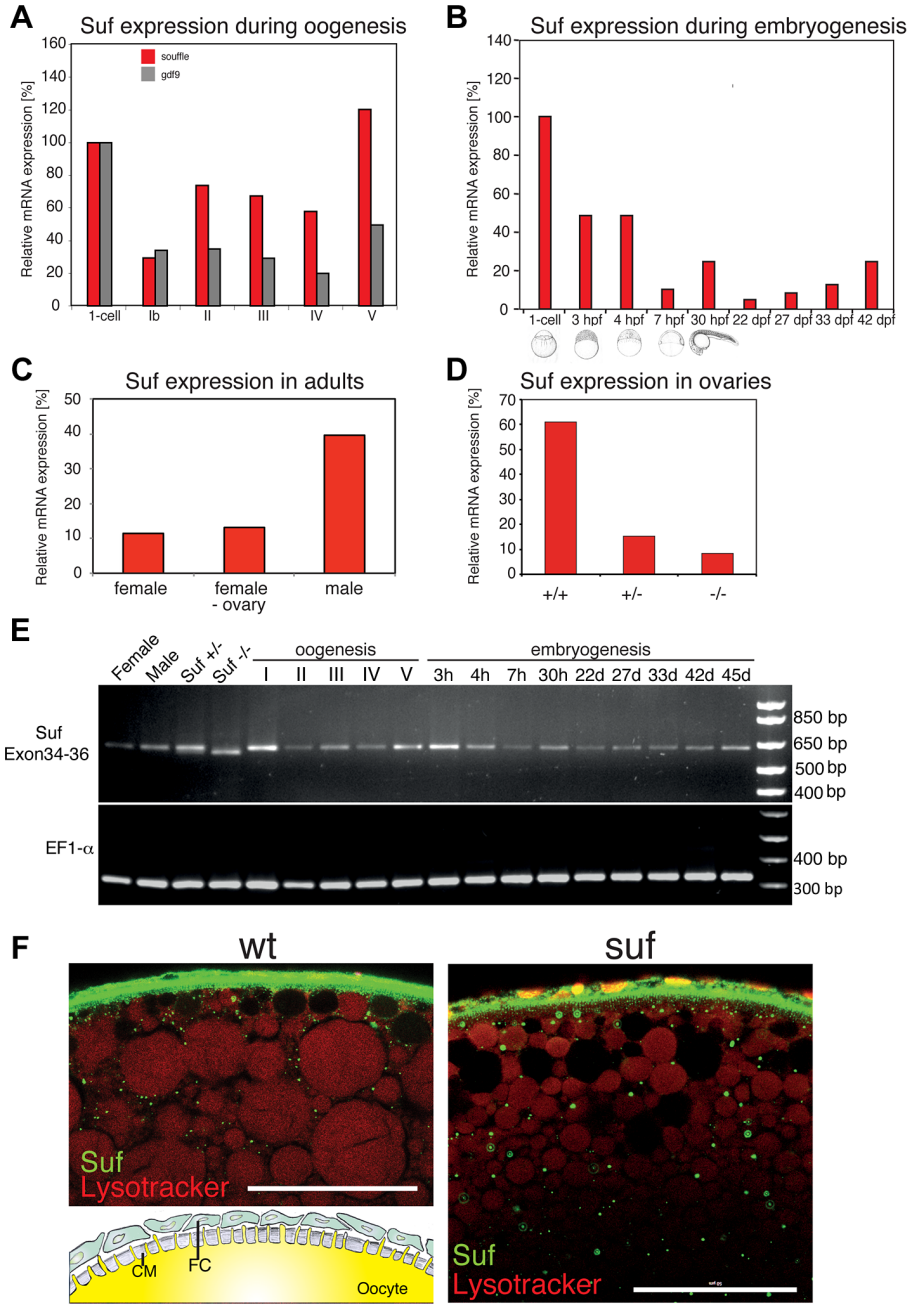
Suf/Spastizin expression analysis. (A) Real-time PCR of *suf*/*spastizin* (red) and *gdf9* (grey) mRNA during oogenesis relative to expression in the one-cell embryo normalized to *gapdh* and *odc1* mRNA. *Gdf9* is exclusively expressed in germ cells, which acts as a positive control for the purification of oocytes particularly in stage Ib oocytes, which are frequently contaminated with somatic follicle cells [111]. (B) *suf*/*spastizin* mRNA levels during embryogenesis, (C) in sexually mature adults, (D) and ovaries of different genotypes. (E) Conventional RT-PCR showing the expression of exon 34–36 (610 bp) of *suf*/*spastizin* mRNA at selected stages (h: hours post fertilization; d: days post fertilization). A lack of exon 35 is predicted to amplify a 413 bp product, which is not observed at any stage of oogenesis or embryogenesis. A longer run of this gel highlighting the 25 bp shorter transcript caused by the change of the splice donor in the mutant controls is shown in Figure S2. (F) Confocal section of wild-type (wt) and mutant (*suf*) stage III oocytes labeled with Suf/Spastizin antibody (green) and counterstained with lysotracker (red). Scale bar: 50 µm. Inset in wt panel shows the architecture of the oocyte cortex with the cytoplasm of the oocyte (yellow) forming microvilli crossing the acellular chorion membrane (CM; grey). The oocyte is surrounded by a layer of follicle cells (FC; green) [modified after 94].

Hence, we investigated whether the *suf*/*spastizin* gene is expressed at the appropriate time to control oogenesis in zebrafish. Real-time PCR analysis of isolated follicles at selected stages showed that *suf*/*spastizin* mRNA is expressed during oogenesis with a slight increase at the onset of vitellogenesis (stage II, for staging see Figure 1A) and another increase after ovulation (stage V) (Figure 2A). This expression profile is consistent with a role of *suf/spastizin* during zebrafish oogenesis.

During embryogenesis, *suf*/*spastizin* mRNA decreased after 4 hpf (hours post fertilization) similar to other maternal genes (Figure 2B). At 30 hpf *suf*/*spastizin* showed a small peak of expression. Later during larval stages, the expression steadily increases consistent with microarray data in the Espresso database (http://zf-espresso.tuebingen.mpg.de; Unigene ID: Dr.21642). To analyze sex-specific expression of *suf/spastizin*, we compared mRNA levels in whole females, females without ovaries and males. Contrary to its specific phenotype in the oocyte, *suf*/*spastizin* was strongly expressed in males and even outside the female germline suggesting that it also acts in somatic cells (Figure 2C).

The higher expression of Suf in males can be explained by two non-exclusive reasons. A simple explanation might be technical, *i.e.* the genes *gapdh* and *odc1*, which we used to normalize mRNA levels, may be differentially expressed between males and females as reported for *ef1α* [38]. Alternatively, Suf is indeed higher expressed in males, possibly in one of the non-reproductive organs with sex-specific gene expression such as the brain or the liver [39]. However, the *p96re* allele clearly demonstrates that Suf is required in the oocyte, but does not exclude that it has a critical role in other organs, which we did not notice.

Since we did not observe a mutant phenotype outside the germline, we addressed whether the *p96re* mutation causes a complete loss-of-function null-allele or forms a hypomorph. Comparison of *suf*/*spastizin* mRNA levels between +/+, +/− and −/− ovaries showed a strong reduction after loss of one *suf*/*spastizin* copy in heterozygous adults, but no phenotype (Figure 2D). By contrast, −/− mutant females showed only a minor reduction of mRNA compared to +/− heterozygotes, but eggs displayed the mutant phenotype. To examine whether an alternatively spliced *suf*/*spastizin* mRNA hides a potential zygotic mutant phenotype in other tissues or in males, we analyzed the expression of exon 35 carrying the mutation during zebrafish embryogenesis and oogenesis. We did not observe a shorter transcript lacking exon 35, which would generate a 413 bp product (Figures 2E and S2A). However, in hetero- or homozygotes we detected the predicted 25 bp shorter transcript consistent with the mutant splice donor. Since *suf*/*spastizin* expression at the mRNA level was not completely eliminated, the residual protein might have sufficient activity to compensate for Suf/Spastizin requirement in somatic cells. To analyze Suf/Spastizin protein expression, we generated an antibody against the zebrafish protein (Figure S2B) and compared mutant and wt oocytes in whole-mount immuno-stainings. Although mRNA levels were strongly reduced in mutant ovaries, Suf/Spastizin protein still sufficiently accumulated during oogenesis and localized to the membrane and cytoplasmic vesicles in wt as well as mutant oocytes (Figure 2F). Taken together, Suf/Spastizin was expressed during zebrafish oogenesis, but was also present outside of the germline.

### *souffle*/*spastizin* mutants accumulate Rab11b-positive vesicles

**Figure 3:**
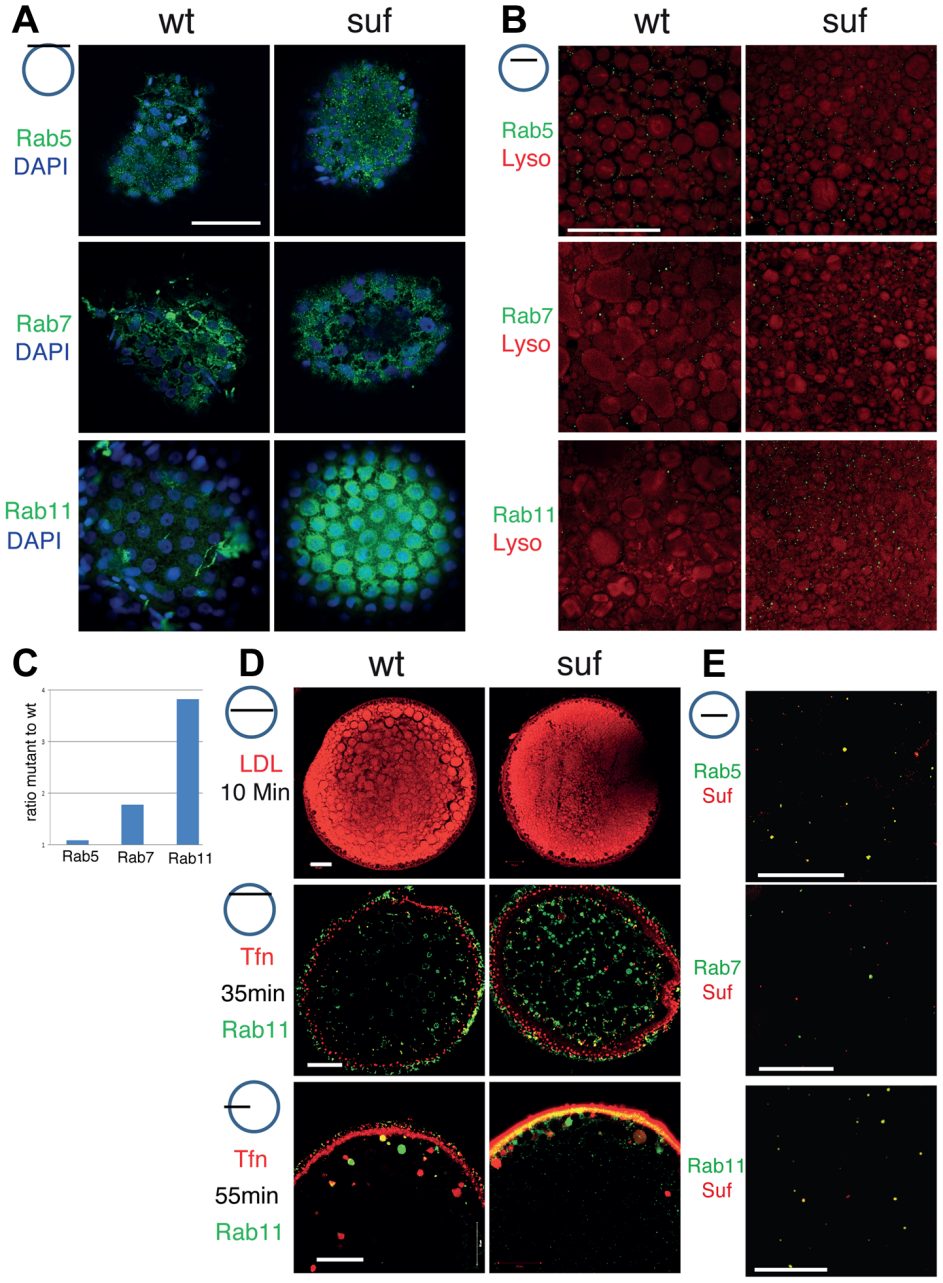
Comparison of endosomal compartments in wild-type and *suf*/*spastizin* mutant oocytes. Small icon next to figures indicates the level of the optical section (black line) in the oocyte (blue circle). (A) Surface view of immuno-labeled stage III oocytes showing Rab5, Rab7 and Rab11 (green). The nuclei of the surrounding follicle cells are labeled with DAPI (blue). Note that Rab11-positive staining accumulates in patches below the layer of follicle cell nuclei. Scale bar: 50 µm. (B) Optical section of stage III oocytes showing no change in the number of Rab5, a slight increase of Rab7 and a strong increase of Rab11 positive vesicles. The oocyte cytoplasm was counterstained with the lysosomal marker Lysotracker (red) labeling yolk globules. Scale bar: 50 µm. (C) Summary of the results of panel B quantified in Figure S3. The bars display the ratio of Rab-positive foci in mutant oocytes to wild-type. Equal numbers correspond to one fold (baseline). (D) Functional analysis of the endosomal trafficking routes by cargo assay. LDL (red) as a marker for the degradative route accumulates within 10 min in fragmented lysosomes. Transferrin (Tfn; red) as a recycling cargo shows no difference between wt and mutant oocytes after 35 (middle) or 55 min (lower panel) pulse/chase. Note that Transferrin accumulates in a Rab11b-negative compartment. Scale bar: 50 µm. (E) In wt oocytes, Rab5 (green; upper panel) colocalizes with Suf/Spastizin (red), but Rab7 does not overlap (middle panel). Suf/Spastizin (red) and Rab11b (green) colocalize predominantly, but independent localization of Rab11 and Suf is also observed (lower panel). Scale bar: 50 µm.

In tissue culture cells, the FYVE domain of Suf/Spastizin interacts with the endosomal lipid PI3P indicating a role in endosomal trafficking [21]. Moreover, in human and mouse cells Spastizin binds to the novel AP5 complex regulating endosomal transport [22], [26], [27], [40]. To examine genetically in zebrafish oocytes, whether Suf/Spastizin is involved in endocytosis during oogenesis, we compared endosomal compartments between wt and mutant. The gross morphology of oocyte vesicles showed no difference in early endosomes (Rab5) or late endosomes (Rab7) (Figure 3A) [41], [42]. In contrast, Rab11b-positive recycling endosomes showed a remarkable transformation of their tubular shape in wt to patches accumulating below the nuclei of the surrounding follicle cells in mutant oocytes (Figure 3A) [43].

To quantify the defect in *suf*/*spastizin* mutants, we counted Rab positive foci in optical sections in deeper layers of the oocyte cytoplasm about 50 µm below the cortex, where single, small vesicles are easier to discriminate than the large compartments at the cortex (Figure 3B, C). Rab5 (early endosomes) showed no significant change (1.09 fold; p = 0.35; Figures 3C and S3A), whereas Rab7 positive endosomes increased moderately (1.78 fold; p = 0.0001; Figures 3C and S3B). However, Rab11 staining increased dramatically (3.82 fold; p = 0.0001; Figures 3C and S3C) confirming the initial observation that Suf/Spastizin controls trafficking of recycling endosomes in zebrafish oocytes. Consistent with previous reports in cell culture and mouse mutants [22], [25], we also discovered in zebrafish that smaller lysosomes accumulated in *suf*/*spastizin* oocytes, possibly preventing yolk proteolysis in the egg and causing the opaque cytoplasm phenotype (Figure 3B).

To functionally analyze during oogenesis whether the observed accumulation of the compartment-specific Rab proteins reflects a defect in the corresponding transport route as described for tissue culture cells, we established cargo trafficking assays in the zebrafish oocyte. LDL follows the degradative transport route to lysosomes and the yolk-receptor belongs to the LDL-receptor superfamily [44], [45]. Adding fluorescent LDL to the culture medium labeled yolk globules in zebrafish oocytes, which correspond to lysosomes of somatic cells [46]. However, we observed no difference in the LDL transport to wt or mutant lysosomes, which were also identified by a characteristic black halo after fixation (Figure 3D, also visible in Figure 3B) suggesting that transport along the degradative pathway is not disrupted.

Transferrin follows the recycling route [47]–[49]. The zebrafish Transferrin-receptor is maternally expressed and the Transferrin recycling assay was previously also applied to *Xenopus* oocytes [50], [51]. Notably, contrary to the accumulation of Rab11 vesicles, fluorescent Transferrin did not accumulate in *suf*/*spastizin* oocytes (Figure 3D). More interestingly, we found rare overlap of Transferrin with Rab11 suggesting that the Rab11b antibody and Transferrin label different compartments in zebrafish oocytes.

To analyze whether Suf/Spastizin directly controls the trafficking of endosomes or whether the accumulation of Rab11b-positive vesicles is a secondary defect, we investigated Suf/Spastizin and Rab colocalization in the oocyte. Although early endosomes did not show a defect in *suf*/*spastizin* mutants, we also discovered colocalization of Suf/Spastizin with Rab5 as shown in the mouse [22], but not with Rab7 (Figures 3E, S4A and S4B). Although Rab11b and Suf/Spastizin mostly overlapped in their localization, some Rab11b vesicles were negative for Suf/Spastizin (Figures 3E and S4C). This finding suggests that Suf/Spastizin might not be involved in all processes regulated by Rab11b in the zebrafish oocyte. In summary, the loss of Suf leads to an accumulation of Rab11b positive vesicles, which in zebrafish oocytes are not involved in Transferrin recycling.

### Suf/Spastizin is not required during meiosis, but during mitosis

**Figure 4:**
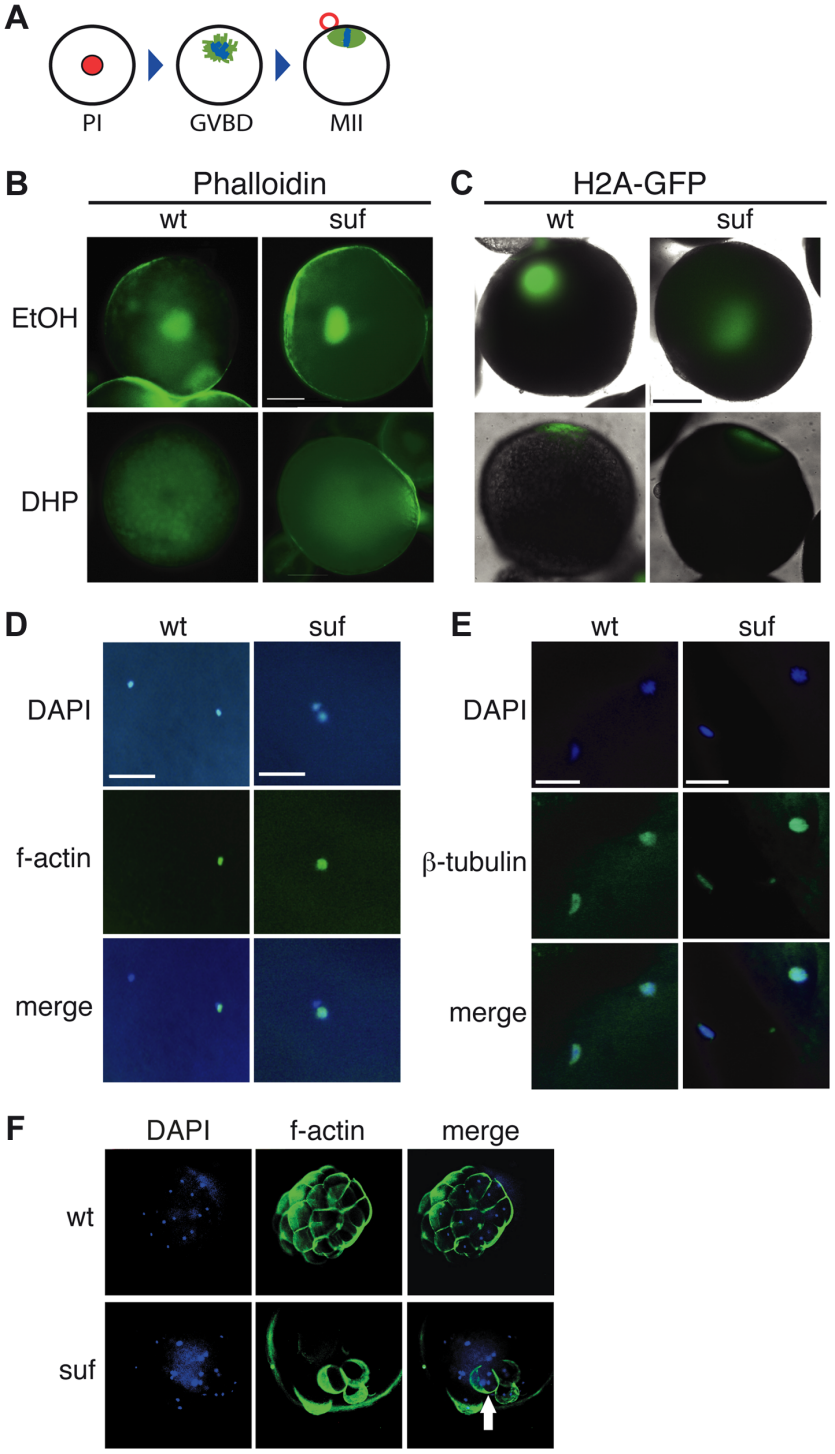
Suf is not essential for meiosis, but mitosis. (A) Steps of meiotic maturation. Zebrafish oocytes are arrested in prophase I (PI) of the first meiotic cell cycle indicated by the huge germinal vesicle (red). Maturation initiates with germinal vesicle breakdown (GVBD) and the first meiosis leads to the formation of a polar body (red circle). The egg arrests again in metaphase of the second meiotic cell cycle (MII), ready to be fertilized. (B) Stage III oocytes treated with carrier (EtOH) or maturation-inducing hormone (DHP). The germinal vesicle is highlighted with Phalloidin (green) showing no difference between wt and mutant. Scale bar: 100 µm. (C) Living stage III oocytes from wt and *suf/spastizin* mothers treated with carrier or DHP. The chromatin is highlighted by a Histon2A-GFP transgene (green) showing that GVBD occurs at the same time in mutant oocytes as in wt, whereas the cytoplasm stays opaque in *suf*/*spastizin* oocytes. Scale bar: 100 µm (D) *suf*/*spastizin* oocytes form a polar body. *In vitro* matured oocytes from wt and mutants stained with DAPI (blue) to highlight the nuclei and f-actin (green) to mark the polar body. Scale bar: 25 µm. (E) *suf*/*spastizin* oocytes arrest in metaphase II. Confocal images of β-tubulin (green) highlight the spindle in wt and mutant oocytes, which is located in the lower left corner, whereas the polar body is in the upper right corner. Scale bar: 25 µm. (F) Suf/Spastizin is required for cytokinesis. Embryos at 32-cell stage from heterozygous wt (top panel) or homozygous (bottom panel) *suf*/*spastizin* mothers with labeled nuclei (DAPI; blue) and plasma membranes (Phalloidin; green). In contrast to wt, few eggs from mutant mothers initiate cell division after fertilization, but then show cells with multiple nuclei (arrowhead).

Since recycling was not disrupted in *suf* oocytes, we examined additional processes requiring recycling endosomes during oogenesis. Endosomal recycling is involved in meiotic maturation in *C. elegans* [52] and *Xenopus laevis* [50], which in teleost oocytes is induced by the progestin 17α,20β-dihydroxy-4-pregnen-3-one (DHP) [53]–[55]. Staining of the germinal vesicle with fluorescent phalloidin revealed that *suf*/*spastizin* oocytes underwent GVBD (germinal vesicle breakdown) in response to DHP (93%, n = 28) similar to wild-type oocytes (82%, n = 17), whereas ethanol-treated control-oocytes retained their germinal vesicle (suf: 64%, n = 11; wt: 57%, n = 28) (Figure 4A, B). This finding was also confirmed by following the dynamics of GVBD *in vivo* with a transgenic H2A-GFP (Histone2A-GFP) reporter line [56] (Figure 4C). We also examined polar body extrusion (Figure 4D) and spindle formation (Figure 4E) during oocyte maturation, but observed no difference between wt and mutant. These results show that Suf is not required for meiotic maturation in the zebrafish oocyte.

In eukaryotes, recycling vesicles and Rab11 are involved in cytokinesis during mitosis [reviewed in 57], [58]–[60]. Consistently, after RNAi depletion of the Suf-homolog FYVE-Cent in HeLa cells, a cytokinesis defect was observed [21]. To analyze cytokinesis in embryonic cells with defective maternal Suf protein, we examined embryos from *suf* mutant mothers. We labeled the cell cortex of 32-cell embryos with Phalloidin and their nuclei with DAPI (Figure 4F). Whereas embryos from wt mothers showed one nucleus per cell, age-matched embryos from *suf* mutants exhibited multinucleated cells. This result suggests that the maternally controlled cell cycles require Suf/Spastizin similar to the cytokinesis defect discovered in HeLa cells. However, these results did not conclusively provide evidence for a role of Suf in endosomal recycling of zebrafish oocytes.

### Rab11 and Souffle/Spastizin colocalize in oocytes on cortical granules

Cell culture studies implicate Rab11 endosomes in additional transport processes besides recycling [reviewed in 61] *e.g.* Rab11 localizes on secretory vesicles of mammalian cells [62]. Yeast, tissue culture cells and *C. elegans* oocytes require Rab11 for exocytosis of secretory vesicles [63]–[67], which are also labeled by Rab11b [43]. Moreover in epithelial cells, Rab11a and −11b localize to distinct compartments [68].

In oocytes of many organisms including humans, secretory vesicles are also designated cortical granules [reviewed in 69], [70]. They are most similar to large, dense-core vesicles found in secretory cells in humans, *e.g.* neurons or pancreatic β-cells [71]–[73]. The most prominent cargoes of cortical granules are carbohydrates, which after secretion increase their volume by hydration leading to chorion elevation and thereby create the perivitelline space between oocyte and chorion. As in mammals, cortical granules are secreted after fertilization during a process termed “cortical reaction” and the induced chorion elevation is important to inhibit lethal polyspermy and mechanical damage to the embryo [reviewed in 69], [70].

**Figure 5:**
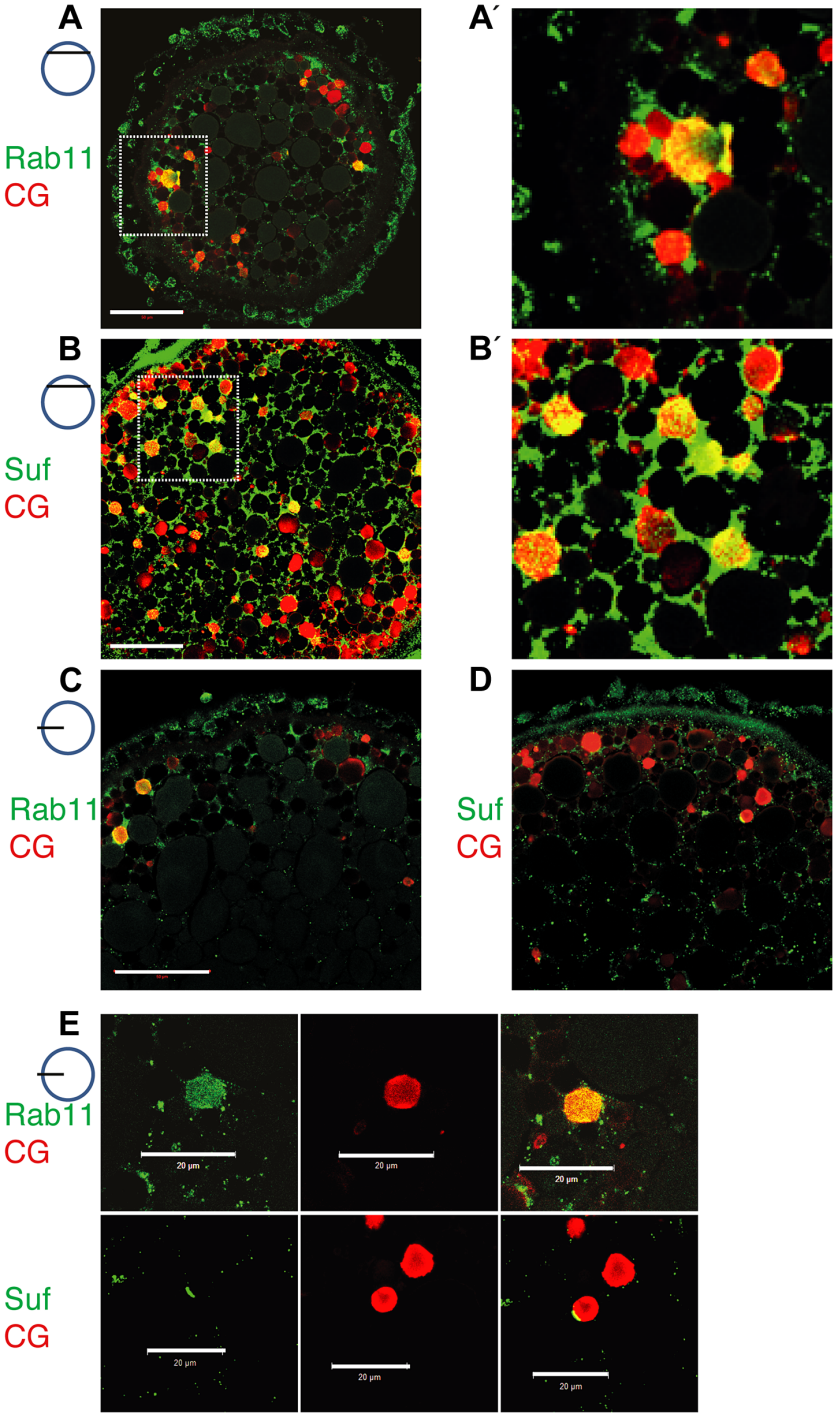
Suf/Spastizin and Rab11b colocalize on cortical granules. Optical sections of stage III oocytes showing the localization of (A, A′) Rab11b (green) or (B, B′) Suf/Spastizin (green) on cortical granules (CG) labeled with MPA-lectin (red). Scale bar: 50 µm. Stippled boxes in A, B highlight magnified area in A′, B′. (C–E) Rab11b and Suf/Spastizin colocalize in central, optical sections on cortical granules. Confocal section showing colocalization of Rab11b (green) (C) or Suf/Spastizin (green) (D) on cortical granules (red) in wild-type oocytes. (E) Upper row: Single channels of Rab11b (green; left panel) and MPA-lectin (red; center panel) showing localization of Rab11b on cortical granules (yellow; right panel). Lower row: Single channels of Suf/Spastizin (green) on cortical granules (red) forming a polarized microdomain on the granule membrane (yellow). Scale bar: 20 µm.

To determine in fish oocytes, whether Rab11b marks secretory granules in fish oocytes, we double-labeled them with the cortical granule marker MPA-lectin [74]. Rab11b colocalized with MPA-lectin identifying the Rab11 vesicles of the zebrafish oocyte as secretory, cortical granules (Figure 5A, A′). Moreover, Suf/Spastizin protein also colocalized with MPA on cortical granules (Figure 5B, B′). Since not all MPA-vesicles were positive for Rab11b or Suf, we hypothesized that colabeling is only observed at the vesicle surface. In contrast, if the optical section is more central in the vesicle, the green Rab11b or Suf-signal was encompassing the luminal MPA-positive cargo. Since Suf was highly enriched in the cortex and hence, colocalization might be caused by protein abundance, we analyzed optical sections in deeper layers of the oocyte with less Suf signal (Figure 5C–E). Indeed, Rab11b and Suf mostly localized outside the granule-lumen (Figure 5C, D). Higher magnifications showed that Rab11 in general stains the entire surface of the vesicle (Figure 5E, upper row), whereas Suf was also found to be restricted to extraluminal microdomains (Figure 5E, lower row). Taken together, the colocalization of Suf with Rab11b and MPA-lectin supports a role of Suf/Spastizin in the formation of cortical granules during zebrafish oogenesis.

### Souffle/Spastizin controls cortical granule maturation during oogenesis

**Figure 6:**
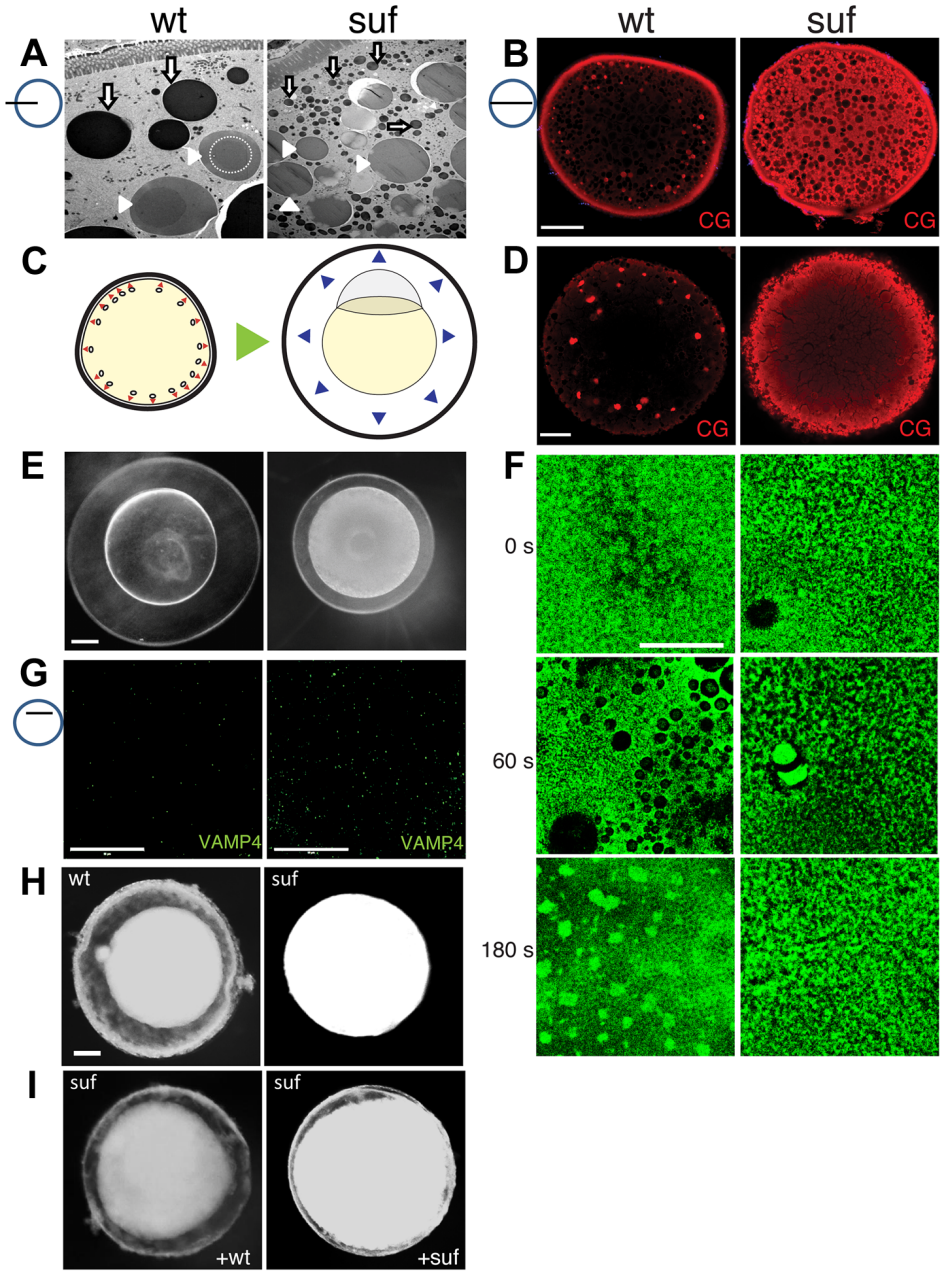
Suf/Spastizin controls cortical granule maturation. (A) Electron micrographs of high-pressure frozen stage III oocytes. Lysosomes (white arrows) are fragmented in *suf*/*spastizin* mutants. Cortical granules (white arrowheads) show a dense-core (dashed circle), which is lost in mutant oocytes. Note, that we consistently generated wrinkles visible as black lines in mutant cortical granules, which indicate sectioning artifacts, but suggest a different composition. Scale bar: 10 µm. (B) Optical section of stage III oocytes showing the accumulation of cortical granules stained with MPA-lectin (red) in *suf*/*spastizin* mutant mothers. Scale bar: 50 µm. (C) Scheme visualizing the exocytosis of cortical granules (red arrowheads, left panel) after egg activation, which leads to chorion elevation (blue arrowheads, right panel) at the beginning of embryogenesis. (D) Cortical granules labeled with MPA-lectin (red) are released after activation in wt, but not in mutants. Scale bar: 50 µm. (E) Fertilized embryos 30 mpf (min post fertilization) from +/− (left panel) and −/− *suf*/*spastizin* mutants (right panel). Note the decreased elevation of the chorion in mutants. Scale bar: 100 µm. (F) Actin cortex of stage V eggs stained with phalloidin (green) at 0, 60 and 180 s after activation. Note the fusion of vesicles in wt eggs after 60 s, visible as black holes in the actin meshwork, and the formation of actin patches at 180 s after exocytosis completion. Scale bar: 50 µm. (G) VAMP4 (green) labels immature secretory granules and accumulates in *suf*/*spastizin* mutant oocytes. Scale bar 50 µm. (H) Stage III oocytes from +/− (wt) or −/− *suf/spastizin* mutants (mut) after 12–16 h incubation in L-15 medium. Scale bar: 50 µm (I) Stage III oocytes from −/− *suf/spastizin* mutants after injection of plasmid encoding wt or mutant Suf (*p96re* allele). Note that the mutant Suf^p96re^ injected oocytes also show chorion elevation similar to wt Suf injected oocytes.

To analyze the role of Suf/Spastizin in cortical granule formation during oogenesis, we compared wt and mutant oocytes using electron microscopy. More cortical granules were visible in *suf*/*spastizin* oocytes (Figures 6A and S5A). Interestingly, their electron dense core was not visible, which is a remarkably strong phenotype compared to other factors involved in dense-core vesicle maturation [75]. We also noted more and smaller lysosomes consistent with the results of the lysotracker staining. To verify an increase in cortical granules, we stained oocytes with MPA-lectin [74]. Wild-type zebrafish oocytes showed a few, large cortical granules in the cytoplasm and numerous, smaller vesicles at the cortex (Figures 6B and S5B). Conversely, mutant oocytes appeared filled with MPA-lectin indicating that Suf/Spastizin is involved in cortical granule formation during oogenesis.

The observed additional granules in zebrafish mutants could be generated by two alternative mechanisms. Either Suf/Spastizin could suppress the formation of granules, which in the mutant leads to an increase of mature, fusogenic vesicles. Alternatively, Suf/Spastizin could regulate the sorting of the MPA epitope, which in the mutant oocyte leads to an increased number of MPA-positive vesicles. At egg activation, cortical granules released their contents during exocytosis leading to chorion elevation (Figure 6C) [76]. Therefore, the first alternative would lead to a stronger chorion-elevation after exocytosis. However, after triggering exocytosis in wt and *suf/spastizin* stage V eggs by H_2_O exposure, we observed no chorion elevation 10 min after egg activation in mutants (Figure 1B). Furthermore, in 30 mpf (min post fertilization) embryos from mutant mothers the chorion was weakly elevated (Figure 6E), although the regular exocytosis process is completed within six minutes after sperm entry [77]. We quantified chorion and embryo diameters to exclude growth defects during oogenesis as a cause for the size differences. Embryo size in wt and mutants were similar, while chorion diameters were reduced in embryos from mutant mothers, which was also apparent in eggs activated without sperm, supporting the idea that chorion elevation is impaired (Figure S5C–G).

These results indicate that Suf/Spastizin controls the sorting of MPA-positive cargo, whose failure leads to the accumulation of immature vesicles in the mutant egg. This hypothesis predicts that immature cortical granules in the mutant egg do not secrete their cargo. Indeed, after activation, mutant eggs still contained numerous cortical granules, whereas wt lost their vesicles close to the cortex with a few left in the inner cytoplasm (compare Figures 6B and 6D).

To directly visualize the fusion of the vesicles, we analyzed the kinetics of exocytosis by labeling cortical actin [74]: 60 s after activation fusing cortical granules generate negatively stained crypts in the wt actin meshwork, whereas *suf* mutants show no vesicle exocytosis (Figure 6F); and 180 s after activation the collapsing crypts form scars of accumulating f-actin in wt oocytes, while the cortex of *suf* mutants did not change. These experiments demonstrate that cortical granules of *suf* mutants are not fusogenic and thus, support the hypothesis that their maturation is controlled by Suf.

In cell culture, immature secretory granules remove specific SNARE proteins from their cytoplasmic surface to acquire the competence to fuse with the plasma membrane [78]–[80]. For instance, depletion of GGA3 in neuroendocrine cells inhibits sorting of VAMP4 away from neuroendocrine secretory granules, which needs to be removed to permit vesicle fusion with the membrane [81]. When we analyzed VAMP4 in zebrafish oocytes, *suf*/*spastizin* mutants accumulated this SNARE protein compared to wt (Figure 6G). These results show that in zebrafish oocytes Suf/Spastizin is essential for the maturation of fusion-competent cortical granules.

Since chorion elevation provided a sensitive read-out for Suf functionality in zebrafish oocytes, we determined whether wt *suf* rescues the mutant phenotype. However, the length of the *suf* gene with almost 8 kb made it problematic to obtain sufficient *in vitro* transcribed RNA for injection and hence, we injected plasmid DNA into oocytes [82]–[84]. Furthermore, we used stage III oocytes, since they can be incubated for longer periods to allow for protein expression than matured stage V eggs. Moreover, zebrafish stage III oocytes also show spontaneous chorion expansion [82]. After 12 h, the majority of wt oocytes elevated their chorion (84.3%±6.9), whereas *suf* mutant oocytes rarely showed a perivitelline space (7.3%±2.9) (Figures 6H and S6A) also after injection of control plasmid (Figure S6B). Wt Suf plasmid partially rescued chorion expansion in mutant oocytes (58.5%±19.0), but the chorion was not elevated to the same extend as in wt (Figures 6I and S6A). Interestingly, when we overexpressed the *p96re* allele, we also observed partial rescue, but at a lower rate (40.0%±13.4) (Figures 6I and S6A). To confirm the rescue with molecular markers, we stained injected oocytes with MPA-lectin and VAMP4. Both markers were reduced in mutant oocytes after injection of plasmid encoding wt Suf or Suf^p96re^, but not after injection of control plasmid (Figure S6B). This result confirms our previous hypothesis that the zebrafish *p96re* allele encodes a hypomorph with reduced activity. Taken together these data demonstrate that Suf controls secretory vesicle maturation probably *via* sorting during zebrafish oogenesis.

### Suf/Spastizin regulates vesicle fission in zebrafish oocytes

**Figure 7:**
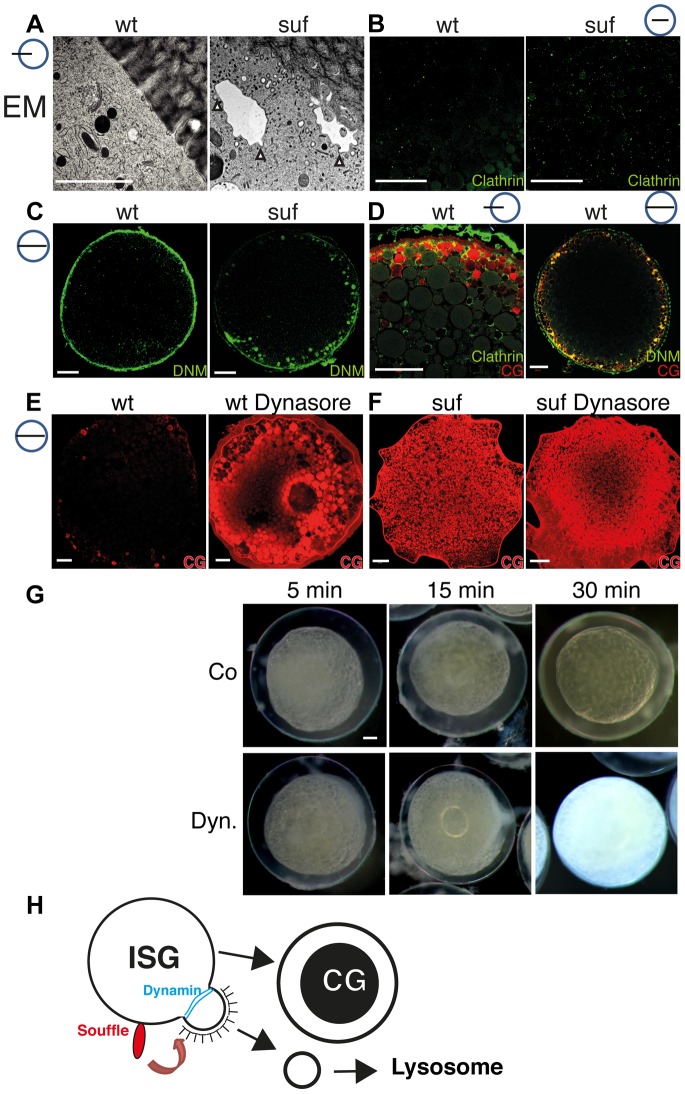
Suf/Spastizin regulates vesicle fission. (A) Electron micrographs of high-pressure frozen stage III oocytes. Cisternae with Clathrin coated buds accumulate in *suf*/*spastizin* oocytes (white arrowheads). Scale bar: 0.5 µm. (B–D) Stage III oocytes labeled with Clathrin (green; B, D) Dynamin (green; C, D) or MPA lectin (red; D). Clathrin and Dynamin localize to a subset of cortical granules. Note the accumulation of Dynamin on vesicles at the cortex in *suf*/*spastizin* oocytes (C). (E, F) Dynasore partially mimics the *suf* defect. Wt oocytes accumulate the cortical granule marker MPA-lectin (red) after treatment with Dynasore (E) similar to *suf* mutant oocytes (F). The huge compartment in the center of the Dynasore treated wt oocyte in panel E shows the germinal vesicle (oocyte nucleus). (G) Cortical granules continuously mature after ovulation. Stage V eggs were incubated with carrier (Co: upper row) or Dynasore (Dyn.; lower row) for 5, 15 or 30 min and then activated with H_2_O. The degree of chorion expansion correlates with Dynasore treatment duration. Note that the complete block of exocytosis also leads to opaque cytoplasm similar to *suf* mutants. (H) Working model for Souffle (red) function on immature secretory granules (ISG). Suf/Spastizin is necessary for the abscission of Clathrin-coated buds. Whereas the compartment matured into secretory, cortical granules (CG) with a dense-core, the Clathrin-coated vesicles enter the endolysosomal transport route probably to lysosomes.

In cell culture experiments, VAMP4 is removed from immature secretory granules after it is sorted into Clathrin-coated buds, which finally pinch off [78], [85], [86]. The localization of Suf to compartmental microdomains also supports a role for Suf in sorting. Consistent with our results, tissue culture experiments previously proposed a role for Suf in sorting [27], which is also a prerequisite for vesicle abscission. To analyze in zebrafish oocytes whether *suf/spastizin* mutants show a defect in vesicle abscission, we returned to the EM analysis and investigated mutants at higher magnification. Interestingly, close to the cortex of mutant oocytes we discovered compartments covered with Clathrin-coated buds, which appeared to not complete budding and abscission (Figures 7A (white arrowheads) and S7A). In wt oocytes these prominent compartments with Clathrin-buds were never observed suggesting that Suf/Spastizin controls a step such as sorting to initiate vesicle budding and fission. To confirm that Clathrin-coated buds accumulate, we compared the localization of Clathrin in wt and mutant oocytes. Clathrin accumulated in mutants corroborating the EM data (Figures 7B, D and S7B, D). The compartments accumulating Clathrin in mutant oocytes looked similar to cisternae formed in the temperature-sensitive Dynamin mutant *shibire* in *Drosophila* [87]. Dynamin exerts the ultimate step after sorting and budding during the fission process [88]–[90]. Indeed, we observed an accumulation of Dynamin on cortical granules in zebrafish *suf*/*spastizin* mutants suggesting that Suf/Spastizin controls a molecular step, which permits Dynamin mediated fission in oocytes (Figures 7C, D and S7C, D). If fission by Dynamin is essential for cortical granule maturation, treatment of zebrafish oocytes with the Dynamin-specific inhibitor Dynasore should mimic the mutant phenotype as long as Suf is required genetically upstream of vesicle fission [91]. Interestingly, wt oocytes accumulated the cortical granule marker MPA similar to mutants after Dynasore treatment (Figures 7E, F and S8A–F). However, we also noted differences such as mature cortical granules in Dynasore-treated wt oocytes, which probably formed during oogenesis before the drug treatment (Figure S8F). Although Dynamin is involved in additional processes as revealed by the Dynasore inhibition, these data confirm that Dynamin mediated fission is required during maturation of secretory granules in the zebrafish egg.

Cortical granule formation was previously considered to be an ongoing process during oogenesis, but whether it continued after ovulation remained unclear [70]. To analyze whether cortical granule maturation still continues in ovulated, fully matured stage V eggs, we treated them for 5, 15 and 30 min with Dynasore and examined chorion elevation. Indeed, the level of chorion elevation corresponded well with the duration of Dynasore treatment (Figures 7G and S8G). Remarkably, the 30 min treatment with Dynasore inhibited chorion elevation completely. Most importantly, Dynasore also reverted the transparency of the egg cytoplasm back to opaqueness similar to *suf* mutant eggs, which we confirmed by MPA-Lectin and VAMP4 staining (Figures 7G and S8H). The transparency of the egg cytoplasm is caused by changes in the crystal structure of yolk globules, which are considered dormant lysosomes [92]–[94]. Theses intriguing results suggest that cortical granule maturation is critical for the function of lysosomal yolk globules during all stages of oogenesis. In summary, our results show that Suf/Spastizin is a key regulator of secretory vesicle maturation during zebrafish oogenesis probably before the fission of Clathrin-coated buds through Dynamin (Figure 7H).

## Discussion

Here we take advantage of zebrafish genetics and its oocytes with their high vesicle trafficking activity to describe a novel role for the SPASTIZIN homolog Souffle. We show that during zebrafish oogenesis Suf/Spastizin is essential for the maturation of secretory granules. Suf/Spastizin colocalizes with Rab11b to an intermediate compartment during the formation of dense-core vesicles. During this process, Suf/Spastizin is necessary for fission from immature secretory granules in zebrafish oocytes.

A striking phenotype in *suf* oocytes is the accumulation of Rab11b positive vesicles. However, in fibroblasts it is unusual that Transferrin does not colocalize with Rab11b and consistently, none of the other phenotypes besides the defect in cytokinesis supports a role for Suf/Spastizin in endosomal recycling. Interestingly, in certain human cell types, secretory cargoes also pass through Rab11-positive endosomes [reviewed in 61]. Moreover, polarized tissue culture cells spatially and functionally separate Rab11a/Transferrin and Rab11b [68], [95]. This remarkable separation of Rab11a and −11b is mostly observed in polarized epithelial cells, which form the Rab11-positive subapical compartment (SAC) or common endosome as a central sorting hub for recycling [reviewed in 96]. Although the zebrafish oocyte is highly polarized along the animal-vegetal axis, polarity at the level of vesicle transport has not been previously described. Therefore, the Rab11b positive compartment in the zebrafish oocyte requires further analysis to confirm its homology to the subapical compartment of human epithelial cells.

Another study performed in rat neuroendocrine PC12 cells implicated Rab11b in the formation of dense-core vesicles [64]. This report showed that Rab11b, but not Rab11a and Rab25 of the Rab11 protein family, are involved in secretion. Moreover, Rab11b was suggested to control the sorting of secretory cargo in neuroendocrine cells. This result in rat tissue culture also provides a molecular mechanism for Suf/Spastizin function most consistent with our results in the zebrafish oocyte. Interestingly, when they repeated their experiments in unpolarized human fibroblasts, *e.g.* HeLa cells, Rab11b showed different effects [64]. This cell-type dependent role of Rab11b in mammals might also explain why the Spastizin homolog was not implicated in regulated secretion before our study, since it was mostly analyzed in unpolarized human fibroblasts [21], [26], [27]. It remains to be determined to which degree our results in zebrafish oocytes are comparable to those in mammalian tissue culture experiments.

The data from human fibroblasts showed that Suf/Spastizin interacts with the novel AP5 complex during the formation of multivesicular-bodies (MVB) and that knock-down of AP5 generates empty MVBs [26], [27]. Consistently, the mouse k.o. demonstrates genetically that Spastizin is critical *in vivo* during this process, which leads to the formation of lysosomes. In zebrafish, we also detected fragmented lysosomes in *suf/spastizin* mutant oocytes. Moreover, mutations in AP5 and SPASTIZIN cause HSP in humans confirming genetically that both proteins are involved in the same process [20], [24]. In addition, Suf/Spastizin also binds to Kif13a [21], which was implicated by tissue culture overexpression experiments to regulate secretion [97]. Since all these studies are carried out in different organisms and different tissues, it is difficult to extract the precise function of Suf/Spastizin.

A major question arising from our study is how the various observed phenotypes could be reconciled. Two alternative scenarios integrate all results: Suf/Spastizin primarily acts in MVB/lysosomes and the maturation defect of secretory vesicles is secondary *e.g.* through retrograde transport to the Trans-Golgi-Network [reviewed in 98] or alternatively, Suf/Spastizin primarily acts in immature secretory granules and the lysosomal defect is secondary *e.g.* through sorting of the mannose-6-phosphate receptors transporting hydrolytic enzymes into lysosomes [reviewed in 71], [99]. Currently, we favor the second model, which is supported by proteins such as AP-3, which play a role in dense core vesicle formation [100] and lysosomal maturation [reviewed in 101]. By contrast, defects in retrograde transport were hitherto not reported to affect the formation of the dense core in secretory granules as in the *suf* mutant, but rather lead to missorting of lysosomal enzymes [reviewed in 98]. This model would also predict that cargo sorted away in immature secretory granules is necessary for homotypic lysosome fusion (Figure 7H) as described in other cells [102]. These open questions make clear that additional studies are necessary to determine the precise role of Suf/Spastizin and whether a defect in lysosome formation or secretion can lead to neuronal degeneration in HSP patients.

In humans, Suf/Spastizin encodes one of the more than 50 loci involved in the neurodegenerative disorder HSP controlling diverse cellular processes [18]. However, regulated secretion was so far not considered. Interestingly, defects in regulated secretion as observed in the zebrafish oocyte may explain some of the HSP symptoms. Cortical granules in the zebrafish oocyte appear identical to large dense-core vesicles (DCV) in neurons [71]–[73], [103], which store neurotrophic factors at the synapse [104]. Neurotrophic factors are responsible for the dynamics and maintenance of synaptic connections during long-term potentiation [105], [106]. In contrast to neurotransmitter vesicles, DCVs need to be transported from the cell body to the synapse. This transport model would resolve why in HSP preferentially the longest axons degenerate from their synapse. In addition, a defect in long-term potentiation would explain why HSP neurons are not maintained and the symptoms become apparent in juveniles and adults, but not in embryos. Investigating the molecular network regulated by Suf/Spastizin in zebrafish oocytes as well as transferring these results to the nervous system as described for Atlastin (SPG3A) in zebrafish [42] and *Drosophila* [107] could provide novel insights into the biochemical etiology of HSP and bring us closer to a therapeutic treatment for patients.

## Materials and Methods

### Ethics statement

Fish were maintained as described [108] in accordance with regulations of the Georg-August University Goettingen, Germany.

### Oocyte and embryo methods

Oocytes were dissected and cultured as previously described [83] and their stage indicated with Roman numerals as published [11]. For *in vitro* maturation and plasmid injection, stage III oocytes were manually dissected and incubated for 1 hr in 60% L-15 medium. After removing damaged oocytes, meiosis resumption was induced by a 90 min-exposure to 1 µg/ml DHP or EtOH as carrier control.

For plasmid injection, stage III oocytes were injected with 1 ng of pCS2+wt-suf or pCS2+mut-suf encoding the *p96re* allele as described [84]. After injection oocytes were incubated for 12 h at 28°C in 90% L-15 medium (0.5% BSA; 100 µg/ml Gentamycin) and then scored for chorion elevation [82].

Suf/Spastizin morpholino injections were performed as previously described [37].

For the trafficking assay, stage III oocytes were incubated with 125 µg/ml Transferrin- Alexa594 (Molecular Probes) in OR2 buffer [108] for 10 and 25 min followed by 30 min of chasing in Transferrin-free OR2 buffer (recycling) or with 10 µg/ml of LDL Dil (Molecular Probes) in OR2 buffer for 10 min (degradation). Then, oocytes were fixed with MEMFA (1 M MOPS pH 7.4, 20 mM EGTA, 10 mM MgSO_4_, 3.7% formaldehyde) after washing twice with OR2 and twice with PBT and stained with antibodies or fluorescent dyes.

For the chorion elevation assay, ovulated stage V eggs were squeezed from gravid females. Eggs were activated by adding E3-medium [108] and imaged at 30 min [108] after activation. Images were used to measure chorion elevation using Fiji software [109].

For Dynasore treatment, live oocytes were collected in OR2 buffer and incubated with 500 µg/ml of Dynasore (Sigma) or the carrier DMSO as control for 1 hr at room temperature. Then, oocytes were washed thrice and processed for immunofluorescence staining.

### Quantitative Real-Time PCR

qRT-PCR on selected stages of oogenesis and embryogenesis was performed as previously described [83].

### Meiotic mapping and genotyping

The described mapping position of the *suf* mutation on chromosome 13 [28] was used for fine mapping as previously published for the *bucky ball* locus [83]. The closest polymorphic markers flanking the mutation are: AL13-10-fw: 5′-GTTCCCACTCAGAGAAACAA-3′, AL13-10-rev: 5′-GTAATGGTGGGGTTTAATGA-3′, AL13-13-fw: 5′-TGCTTAAATTGCAGTTACAATAA-3′, AL13-13-rev: 5′-TGAGATGCGTCTTTAAGTTG-3′. The cDNA sequence of wt and mut *souffle* were submitted to gene bank with the accession numbers KC707919 and KC707920. The *souffle* mutation is registered in the zebrafish Zfin-database (zfin.org) with the ZFIN-ID: ZDB-GENE-070117-691, the gene as: ZDB-GENE-030131-3286 and in the zebrafish genome database at Ensembl (www.ensembl.org) under the gene id: ENSDARG00000040131.

### Phylogenetic analysis

Alignments were performed with CulstalW and the similarity was quantified with vector NTI (Invitrogen). The phylogenic tree was constructed as described [83] with 1000 iterations.

### Tissue culture and western blot

SW480 cells were transfected with 5 µg plasmid using Lipofectamine 2000 (Invitrogen). Cell lysates were separated on a 6% PAA-gel and blotted for 24 h at 40 V on PVDF-membranes in blotting buffer (5% MeOH, 15% western salts). Suf antibody (1∶200) was detected with anti-rabbit HRP.

### Immunofluorescence

Oocytes were dissected from gravid female and fixed with MEMFA buffer for 1 h at room temperature after proteinase K (50 µg/µl) treatment for 3 min. Oocytes were washed thrice with PBT and blocked with PBT containing 2% BSA and 2% Horse serum for 2 h at room temperature. Subsequently, oocytes were incubated with primary antibody: Rab5 (1∶200 SCBT), Rab7 (1∶1000 abcam) [42], Rab11b (1∶500 GeneTex), Suf (1∶200), Dynamin2 (1∶200 GeneTex), Clathrin (1∶200 abcam) and VAMP4 (1∶200 SySy, Goettingen, Germany) in blocking solution overnight at 4°C. Secondary antibodies were added at 1∶200 (Alexa 488 or 594; Molecular probes) in blocking solution overnight at 4°C. For double labeling, oocytes were incubated with Suf-antibody directly labeled with ATTO590 (SySy, Goettingen, Germany) for 2 h at room temperature. The Suf antibody was generated by immunizing two rabbits each with two peptides TEQVKVPAKDRNRE (aa 187–200) and LNKTSTNKGMSKTD (aa 1007–1020) and then purified by affinity-chromatography (Biogenes, Berlin Germany). DNA was stained with 1 µM of Hoechst 33342. Cortical granules were stained with 50 µg/ml MPA Lectin Texas Red (EY Labs Inc.) and lysosomes with 70 nM Lysotracker DND-99 Red (Molecular Probes). Phalloidin staining was performed as described [74].

### Confocal microscopy

Images were captured at room temperature using a LSM780 confocal microscope (Carl Zeiss) with a Plan Apochromat 63×/1.4 NA and 25×/0.8 NA oil-immersion and a digital microscope camera (M27; Carl Zeiss). After washing the oocytes with PBT, yolk was cleared with Murray's solution (Benzylbenzoate (66%)/Benzylalcohol (33%)) during imaging. A multiple wavelength laser was used to visualize red (561) fluorescence, green (488, 405) and blue (405) and images were acquired and processed using ZEN 2011 software (Carl Zeiss).

### Electron microscopy

For high-pressure freezing EM, living oocytes were placed in aluminum platelets of 150 µm depth containing 1-hexadecen [110]. The platelets were frozen using a Leica Em HPM100 high-pressure freezer (Leica Mikrosysteme Vertrieb GmbH, Wetzlar, Germany). The frozen oocytes were transferred to an automatic Freeze Substitution Unit Leica EM AFS2. The samples were substituted at −90°C in a solution containing anhydrous acetone, 0.1% tannic acid for 24 h and in anhydrous acetone, 2% OsO_4_, 0.5% anhydrous glutaraldehyde (EMS Electron Microscopical Science, Ft. Washington, USA) for additional 8 h. After a further incubation over 20 h at −20°C, samples were warmed up to 4°C and washed with anhydrous acetone subsequently. The samples were embedded at room temperature in Agar 100 (Epon 812 equivalent) at 60°C over 24 h. Images were taken in a Philips CM120 electron microscope (Philips Inc.) using a TemCam 224A slow scan CCD camera (TVIPS, Gauting, Germany).

### Statistics

In all experiments, error bars indicate the standard deviation (at least three independent experiments). The statistical significance (p-value) of two groups of values was calculated using a two-tailed, two-sample unequal variance t-test calculated in MS-Excel or www.graphpad.com.

## Supporting Information

Figure S1 Phenotype of embryos after Suf/Spastizin morpholino-injection. Lateral views of embryos 48 h post fertilization (hpf), anterior to the left, after injection of control-morpholino (A) or a Suf/Spastizin specific translation block morpholino (B). The Suf-morpholino causes a twisted tail in 43% of the embryos (left panel), whereas 35% of the embryos show no morphological abnormalities. (C) Quantification of phenotypes.
(TIF)

Figure S2 Suf expression. (A) Expression analysis of exon 35 of *suf* mRNA during oogenesis and embryogenesis. Scheme showing the genomic locus of Suf. Exons 34–36 (orange arrows) and the *p96re* point mutation (red bar) are indicated. Black numbers represent the nucleotide position in the mRNA, green numbers and arrows indicate the position of the primers used to perform RT-PCR, also shown in Figure 2. Primer sequence: exon34-fw 5′-ACGATGGATGAGGATATCCTGG-3′, exon36-rev 5′-CTGGAGACATCAGTGGAGCTCATTTTC-3′. RT-PCR amplifying exon 34–36 (610 bp). Longer run of gel in Figure 2E showing the 25 bp shorter transcript (white arrow) in +/− and −/− ovaries. h: hours post fertilization; d: days post fertilization. EF1-α serves as a loading control. (B) Specificity of polyclonal Suf antibody. Human SW 480 cells were transfected with zebrafish wt Suf and mut Suf^p96re^ and the protein detected by western blotting. To increase the size difference, wt Suf was tagged by a GFP fusion. The antibody recognizes the predicted 314 kD for wt Suf (black arrowhead; Suf: 280 kD + GFP: 34 kD), whereas the mutant Suf^p96re^ shows a band at the predicted size of 250 kD (red arrowhead). Labels on the right indicate the position of the molecular weight markers.
(TIF)

Figure S3 Quantification of Rab staining. (A) Rab5-positive puncta in immuno-labeled stage III oocytes were counted using Image J software. Diagram showing the quantification from wt (left bar) and *suf*/*spastizin* mutant oocytes (right bar). (WT n = 17; suf n = 26; P = 0.3511 NS-not significant). (B) Quantification of Rab7-positive puncta (WT n = 17; suf n = 20; ***, P = 0.0001). (C) Quantification of Rab11 puncta (WT n = 18, suf n = 19, P = 0.0001). Error bars represent standard deviation.
(TIF)

Figure S4 Single channels of Suf and Rab colocalization. Partial colocalization of Suf/Spastizin (red) with Rab-GTPases. (A) Rab5 (green) shows some colocalization with Suf. Venn diagram quantifying Rab5 and Suf costaining. Counting red, green and yellow foci of oocytes (n = 10) reveals that 83±25% Rab5 spots are negative for Suf and 68±16% Suf spots are negative for Rab5. Hence, 17% of the Rab5 positive vesicles contain Suf and 32% of the Suf positive vesicles Rab5. (B) Rab7 shows almost no colocalization with Suf, whereas Suf colocalizes with Rab11 vesicles more frequently (C). Scale bar: 50 µm. Small icon next to figures indicates the level of the optical section (black line) in the oocyte (blue circle).
(TIF)

Figure S5 Suf/Spastizin controls cortical granule maturation. (A) Electron micrographs showing yolk globules and mature cortical granules in wt oocytes (top row). Note the darker dense core in the center of mature cortical granules. *Suf*/*Spastizin* mutant oocytes (bottom row) show immature cortical granules without dense core. Image frame: 2 µm (top, right panel) 10 µm (other panels). (B) *Suf*/*Spastizin* mutants accumulate cortical granules. Confocal sections comparing cortical granules labeled with MPA-Lectin (red) of wt (top) and *suf* mutants (bottom). Small icon next to figures indicates the level of the optical section (black line) in the oocyte (blue circle). Scale bar: 50 µm. (C–G) Quantification of chorion elevation defect in *suf*/*spastizin* mutant embryos and eggs. (C) Small icon indicates distances quantified in bar diagrams of panel D (blue; whole embryo diameter including chorion), E (green; distance between embryo and chorion) and F (red; embryo diameter minus chorion). (D) Quantification of whole embryo size including chorion from +/+ (blue), +/− (red) and −/− (green) mothers at 30 mpf (+/+ n = 37; +/− n = 23; −/− n = 37). (E) Chorion elevation (distance between embryo and chorion). (F) Embryo diameter minus chorion. (G) Diameter of wt (blue) and mutant eggs (red) 30 min after activation in water. Error bars represent standard deviation. Sample size (n-value) is identical for panel D, E and F.
(TIF)

Figure S6 Rescue of chorion elevation defect. (A) Quantification of plasmid injection experiments in Figure 6 H and I showing the number of oocytes with elevated chorions. Error bars represent standard deviation. (B) Morphological phenotype (upper row) of chorion elevation in activated wt (wt + co plasmid) (100%; n = 55), but not in mut oocytes after injection with control DNA (*suf* + Co plasmid) (0%; n = 56). Mutant oocytes injected with plasmid encoding wt Suf (mut + wt Suf) (87.5%; n = 56) or mut Suf^p96re^ (mut + mut Suf^p96re^) (67.9%; n = 53) show chorion elevation. Rescue of MPA-lectin (middle row) and VAMP4 (lower row) accumulation on immature secretory granules after injection of plasmid encoding wt Suf (mut + wt Suf) or mut Suf^p96re^ (mut + mut Suf^p96re^) into mutant oocytes. Scale bars: 50 µm.
(TIF)

Figure S7 Suf/Spastizin is essential for vesicle fission. (A) Accumulation of Clathrin-coated cisternae in *suf*/*spastizin* mutants. Electron micrographs showing cellular compartments from wt (top row) and *suf/spastizin* mutants (lower row). The cortex region is marked by the fenestrated area in some panels, which represents the *zona radiata* forming the chorion membrane after fertilization. Bottom panels show vesicular compartments accumulating Clathrin buds on their membrane in *suf*/*spastizin* mutants. Picture frame: 1 µm (left column) 2 µm (middle and right column). (B–D) Dynamin and Clathrin accumulation on cortical granules of *suf*/*spastizin* mutants. (B) Confocal sections comparing Clathrin localization (green) in wt and *suf*/*spastizin* mutants. (C) Confocal sections comparing Dynamin localization (green) in wt and *suf*/*spastizin* mutant oocytes. Left column shows optical section in the oocyte center. Middle column: optical section of cortex region. Right panel: Cortical section showing the accumulation of Dynamin (green) in mutants. (D) Colocalization of Clathrin (green) or Dynamin (green) on cortical granules (red). Left panel of Clathrin staining shows the cortex region and right panel shows the center of the oocyte. Dynamin panel shows cortex region. Scale bar: 50 µm. Small icon next to figures indicates the level of the optical section (black line) in the oocyte (blue circle).
(TIF)

Figure S8 The Dynamin inhibitor Dynasore mimics the *suf*/*spastizin* phenotype. Cortical granules stained with MPA-Lectin (red) in wild type (A, B) or *suf*/*spastizin* mutants (C, D) after treatment with the carrier DMSO (A, C) or with Dynasore (B, D). The dashed square in panel C indicates the magnification in panel E. (E) In untreated, *suf*/*spastizin* mutants MPA-Lectin (red) foci accumulated on the surface of MPA-negative vesicles similar to the cisternae discovered by EM suggesting that MPA-Lectin cargo is sorted, but not pinched off the compartment. (F) Cortex region of *suf*/*spastizin* oocyte before and after Dynasore treatment. Remarkably, *suf* mutants treated with Dynasore showed cortical granules with weak MPA-Lectin background (white arrowheads), which suggested that Dynasore inhibited sorting and removal of the MPA-Lectin cargo into buds. Small icon next to figures indicates the level of the optical section (black line) in the oocyte (blue circle). Scale bar: 50 µm. (G) Quantification of total egg size shown in Figure 7G. Note the significant size reduction of chorion elevation after 15 min. Error bars represent standard deviation. (H) Cellular marker analysis of ovulated stage V eggs. A 30 min treatment with Dynasore of ovulated eggs leads to an accumulation of MPA-lectin (red) and VAMP4 (green) similar to *suf* mutants. Scale bar: 50 µm.
(TIF)
