# Supplementary figures and images for: Souffle/Spastizin Controls Secretory Vesicle Maturation during Zebrafish Oogenesis
Source: PLoS Genet. 2014 Jun 26;10(6):e1004449. doi: 10.1371/journal.pgen.1004449 (PMC4072560; doi:10.1371/journal.pgen.1004449)

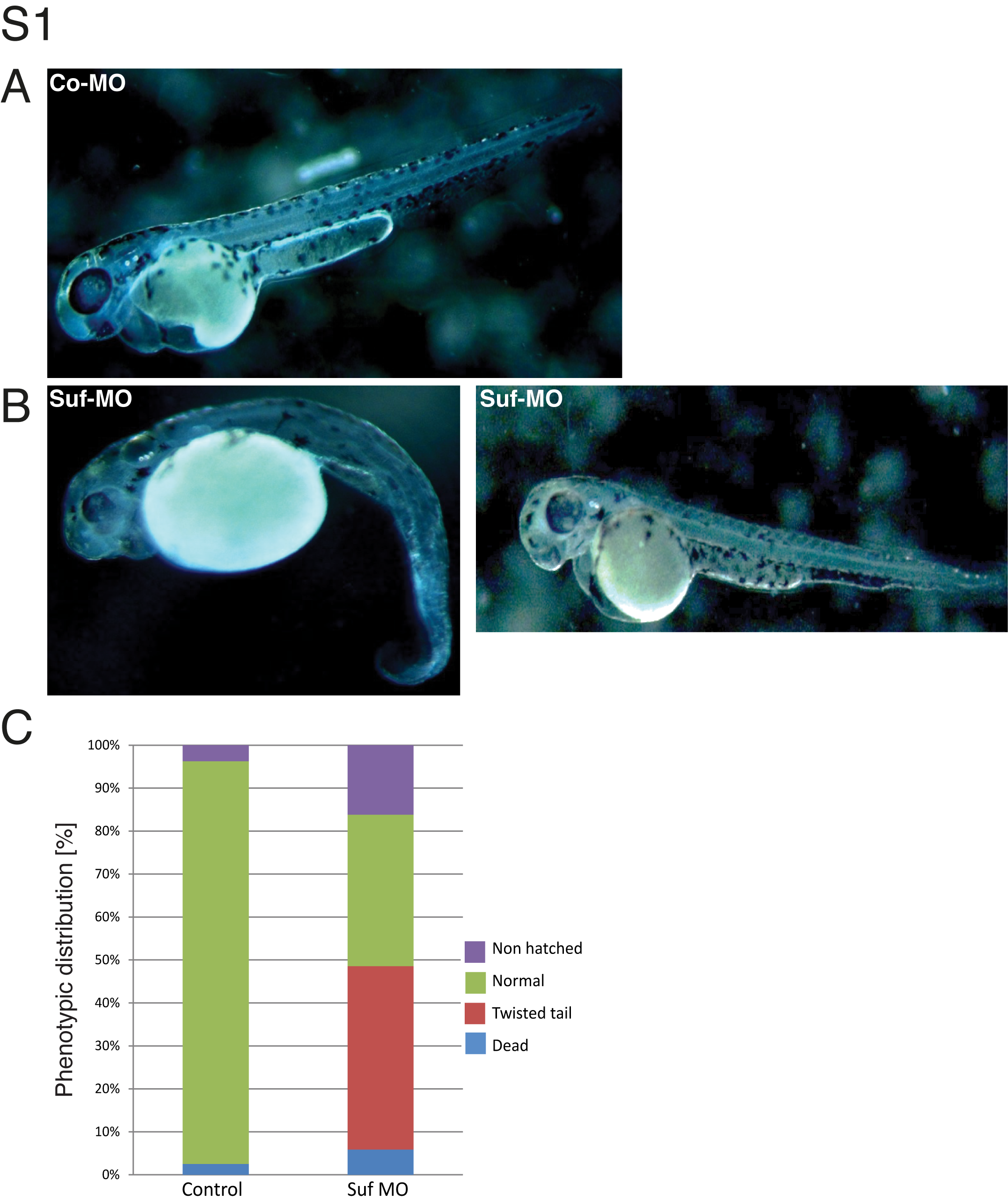

Supplement: Figure S1 — Phenotype of embryos after Suf/Spastizin morpholino-injection. Lateral views of embryos 48 h post fertilization (hpf), anterior to the left, after injection of control-morpholino (A) or a Suf/Spastizin specific translation block morpholino (B). The Suf-morpholino causes a twisted tail in 43% of the embryos (left panel), whereas 35% of the embryos show no morphological abnormalities. (C) Quantification of phenotypes. (TIF) [file pgen.1004449.s001.tif]

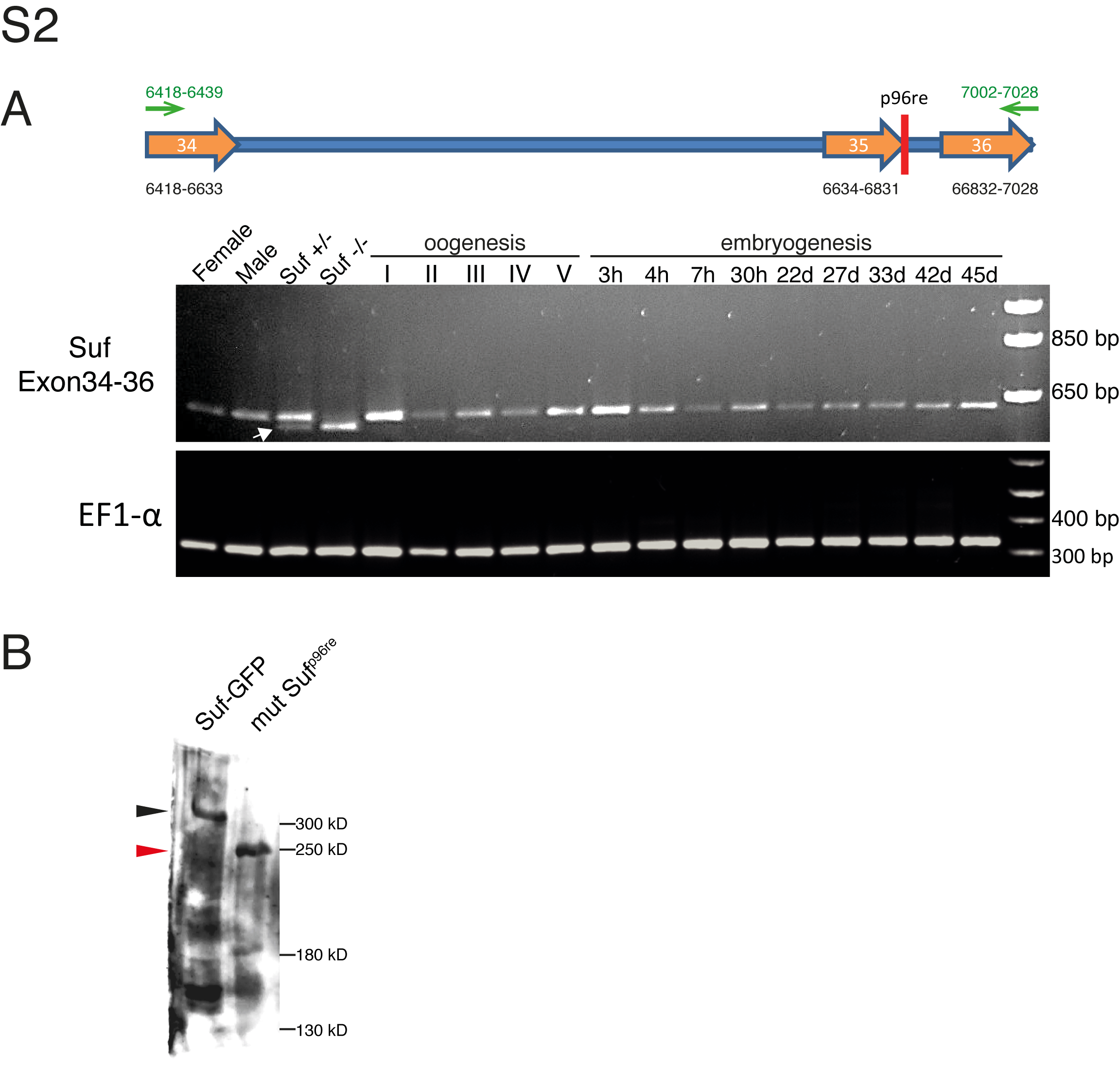

Supplement: Figure S2 — Suf expression. (A) Expression analysis of exon 35 of suf mRNA during oogenesis and embryogenesis. Scheme showing the genomic locus of Suf. Exons 34–36 (orange arrows) and the p96re point mutation (red bar) are indicated. Black numbers represent the nucleotide position in the mRNA, green numbers and arrows indicate the position of the primers used to perform RT-PCR, also shown in Figure 2. Primer sequence: exon34-fw 5′-ACGATGGATGAGGATATCCTGG-3′, exon36-rev 5′-CTGGAGACATCAGTGGAGCTCATTTTC-3′. RT-PCR amplifying exon 34–36 (610 bp). Longer run of gel in Figure 2E showing the 25 bp shorter transcript (white arrow) in +/− and −/− ovaries. h: hours post fertilization; d: days post fertilization. EF1-α serves as a loading control. (B) Specificity of polyclonal Suf antibody. Human SW 480 cells were transfected with zebrafish wt Suf and mut Sufp96re and the protein detected by western blotting. To increase the size difference, wt Suf was tagged by a GFP fusion. The antibody recognizes the predicted 314 kD for wt Suf (black arrowhead; Suf: 280 kD + GFP: 34 kD), whereas the mutant Sufp96re shows a band at the predicted size of 250 kD (red arrowhead). Labels on the right indicate the position of the molecular weight markers. (TIF) [file pgen.1004449.s002.tif]

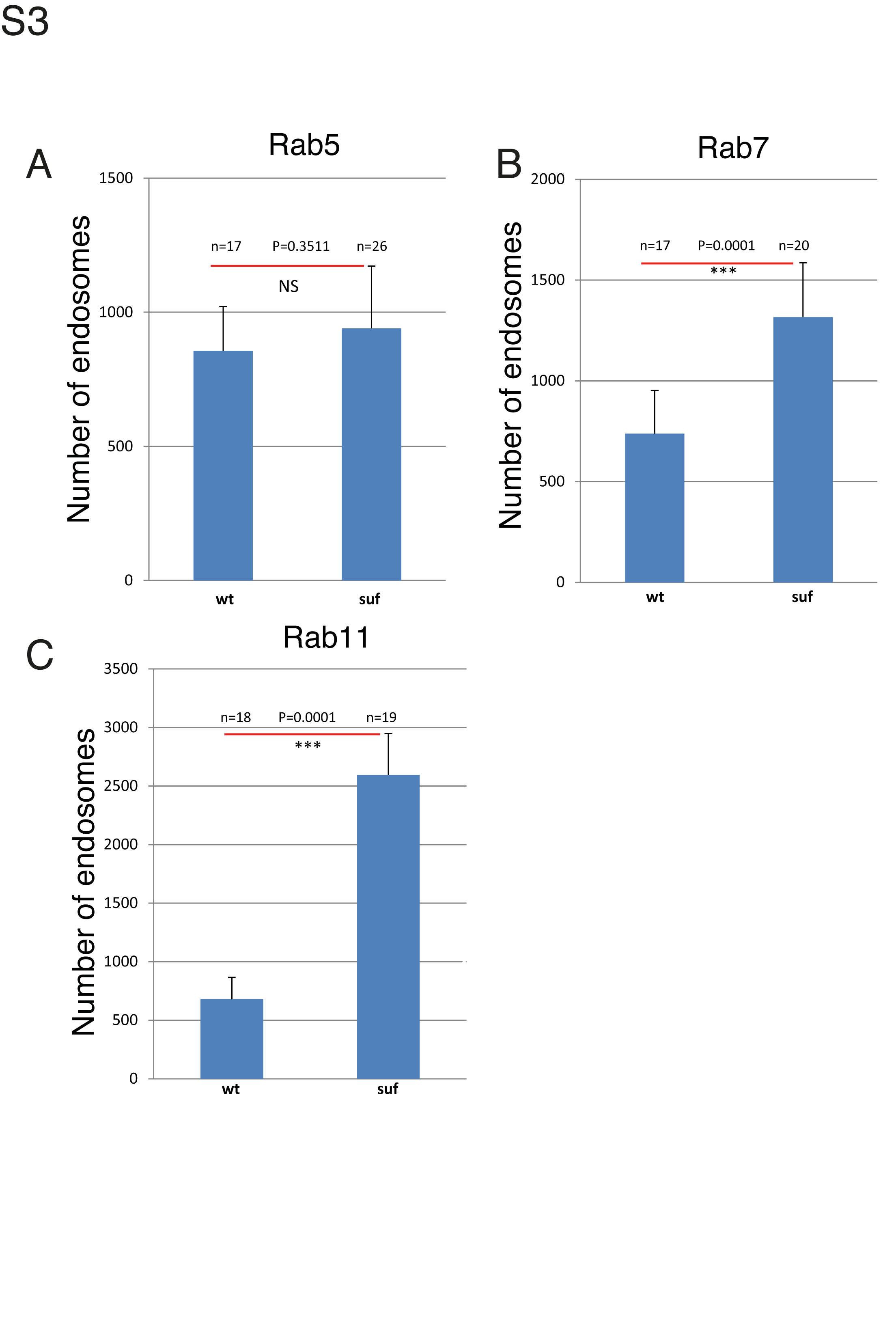

Supplement: Figure S3 — Quantification of Rab staining. (A) Rab5-positive puncta in immuno-labeled stage III oocytes were counted using Image J software. Diagram showing the quantification from wt (left bar) and suf/spastizin mutant oocytes (right bar). (WT n = 17; suf n = 26; P = 0.3511 NS-not significant). (B) Quantification of Rab7-positive puncta (WT n = 17; suf n = 20; ***, P = 0.0001). (C) Quantification of Rab11 puncta (WT n = 18, suf n = 19, P = 0.0001). Error bars represent standard deviation. (TIF) [file pgen.1004449.s003.tif]

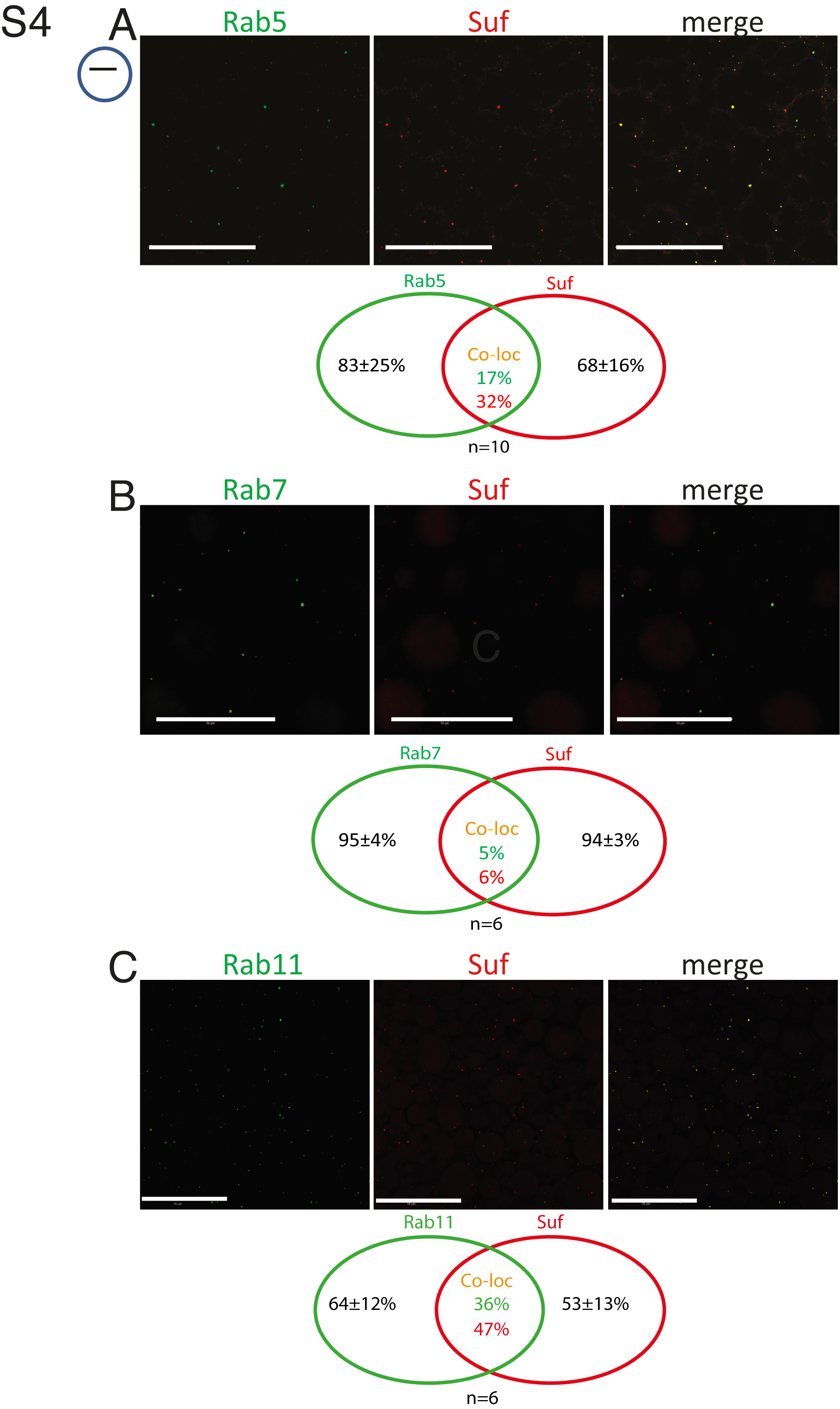

Supplement: Figure S4 — Single channels of Suf and Rab colocalization. Partial colocalization of Suf/Spastizin (red) with Rab-GTPases. (A) Rab5 (green) shows some colocalization with Suf. Venn diagram quantifying Rab5 and Suf costaining. Counting red, green and yellow foci of oocytes (n = 10) reveals that 83±25% Rab5 spots are negative for Suf and 68±16% Suf spots are negative for Rab5. Hence, 17% of the Rab5 positive vesicles contain Suf and 32% of the Suf positive vesicles Rab5. (B) Rab7 shows almost no colocalization with Suf, whereas Suf colocalizes with Rab11 vesicles more frequently (C). Scale bar: 50 µm. Small icon next to figures indicates the level of the optical section (black line) in the oocyte (blue circle). (TIF) [file pgen.1004449.s004.tif]

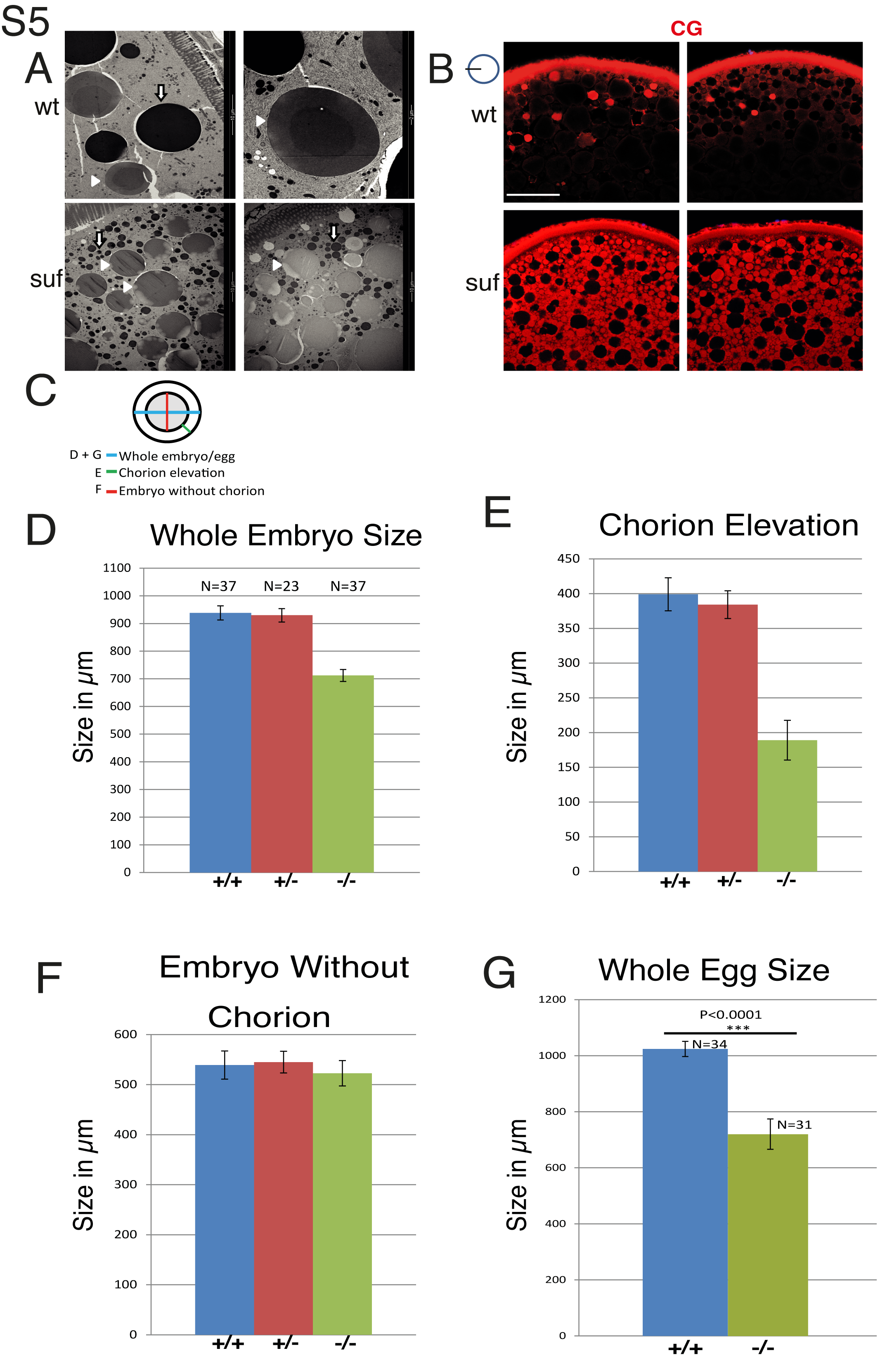

Supplement: Figure S5 — Suf/Spastizin controls cortical granule maturation. (A) Electron micrographs showing yolk globules and mature cortical granules in wt oocytes (top row). Note the darker dense core in the center of mature cortical granules. Suf/Spastizin mutant oocytes (bottom row) show immature cortical granules without dense core. Image frame: 2 µm (top, right panel) 10 µm (other panels). (B) Suf/Spastizin mutants accumulate cortical granules. Confocal sections comparing cortical granules labeled with MPA-Lectin (red) of wt (top) and suf mutants (bottom). Small icon next to figures indicates the level of the optical section (black line) in the oocyte (blue circle). Scale bar: 50 µm. (C–G) Quantification of chorion elevation defect in suf/spastizin mutant embryos and eggs. (C) Small icon indicates distances quantified in bar diagrams of panel D (blue; whole embryo diameter including chorion), E (green; distance between embryo and chorion) and F (red; embryo diameter minus chorion). (D) Quantification of whole embryo size including chorion from +/+ (blue), +/− (red) and −/− (green) mothers at 30 mpf (+/+ n = 37; +/− n = 23; −/− n = 37). (E) Chorion elevation (distance between embryo and chorion). (F) Embryo diameter minus chorion. (G) Diameter of wt (blue) and mutant eggs (red) 30 min after activation in water. Error bars represent standard deviation. Sample size (n-value) is identical for panel D, E and F. (TIF) [file pgen.1004449.s005.tif]

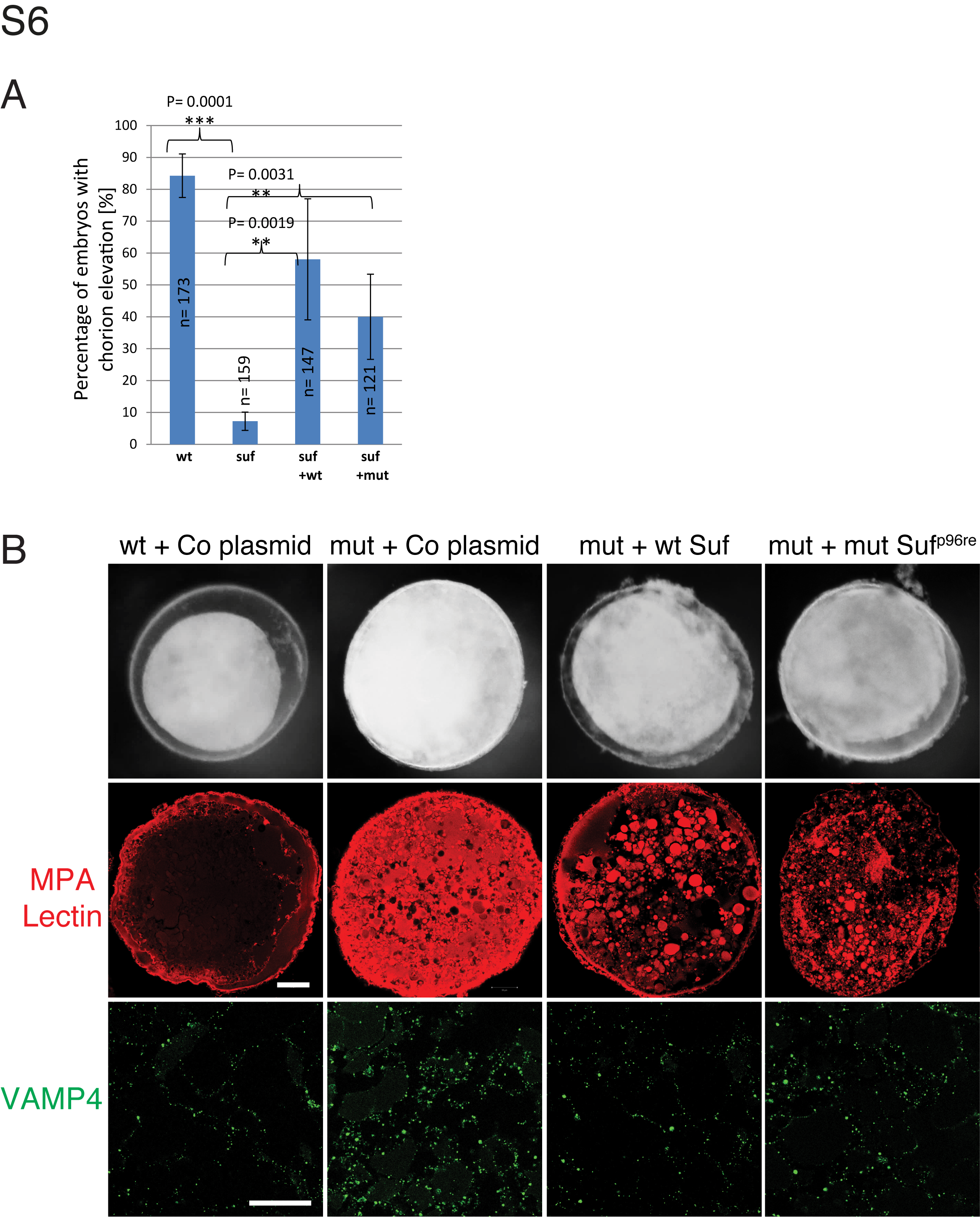

Supplement: Figure S6 — Rescue of chorion elevation defect. (A) Quantification of plasmid injection experiments in Figure 6 H and I showing the number of oocytes with elevated chorions. Error bars represent standard deviation. (B) Morphological phenotype (upper row) of chorion elevation in activated wt (wt + co plasmid) (100%; n = 55), but not in mut oocytes after injection with control DNA (suf + Co plasmid) (0%; n = 56). Mutant oocytes injected with plasmid encoding wt Suf (mut + wt Suf) (87.5%; n = 56) or mut Sufp96re (mut + mut Sufp96re) (67.9%; n = 53) show chorion elevation. Rescue of MPA-lectin (middle row) and VAMP4 (lower row) accumulation on immature secretory granules after injection of plasmid encoding wt Suf (mut + wt Suf) or mut Sufp96re (mut + mut Sufp96re) into mutant oocytes. Scale bars: 50 µm. (TIF) [file pgen.1004449.s006.tif]

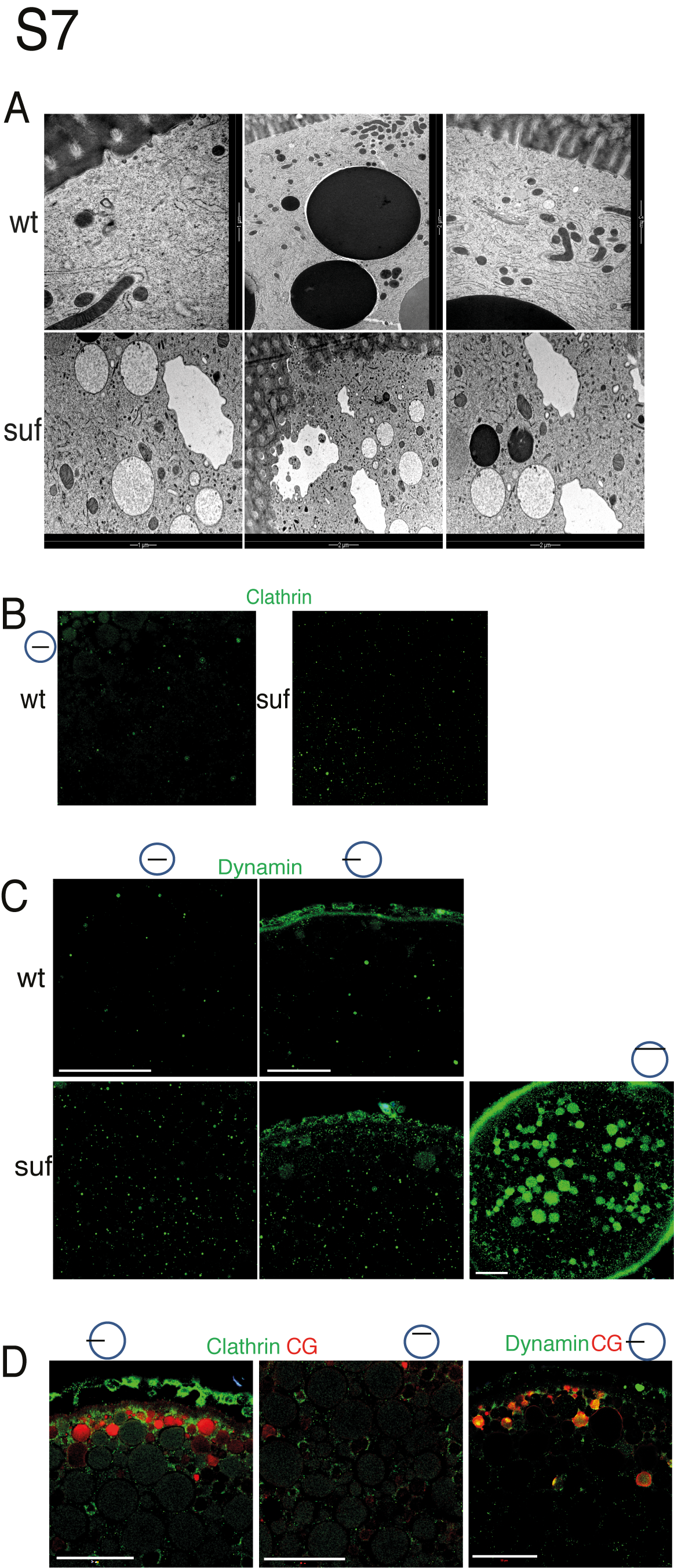

Supplement: Figure S7 — Suf/Spastizin is essential for vesicle fission. (A) Accumulation of Clathrin-coated cisternae in suf/spastizin mutants. Electron micrographs showing cellular compartments from wt (top row) and suf/spastizin mutants (lower row). The cortex region is marked by the fenestrated area in some panels, which represents the zona radiata forming the chorion membrane after fertilization. Bottom panels show vesicular compartments accumulating Clathrin buds on their membrane in suf/spastizin mutants. Picture frame: 1 µm (left column) 2 µm (middle and right column). (B–D) Dynamin and Clathrin accumulation on cortical granules of suf/spastizin mutants. (B) Confocal sections comparing Clathrin localization (green) in wt and suf/spastizin mutants. (C) Confocal sections comparing Dynamin localization (green) in wt and suf/spastizin mutant oocytes. Left column shows optical section in the oocyte center. Middle column: optical section of cortex region. Right panel: Cortical section showing the accumulation of Dynamin (green) in mutants. (D) Colocalization of Clathrin (green) or Dynamin (green) on cortical granules (red). Left panel of Clathrin staining shows the cortex region and right panel shows the center of the oocyte. Dynamin panel shows cortex region. Scale bar: 50 µm. Small icon next to figures indicates the level of the optical section (black line) in the oocyte (blue circle). (TIF) [file pgen.1004449.s007.tif]

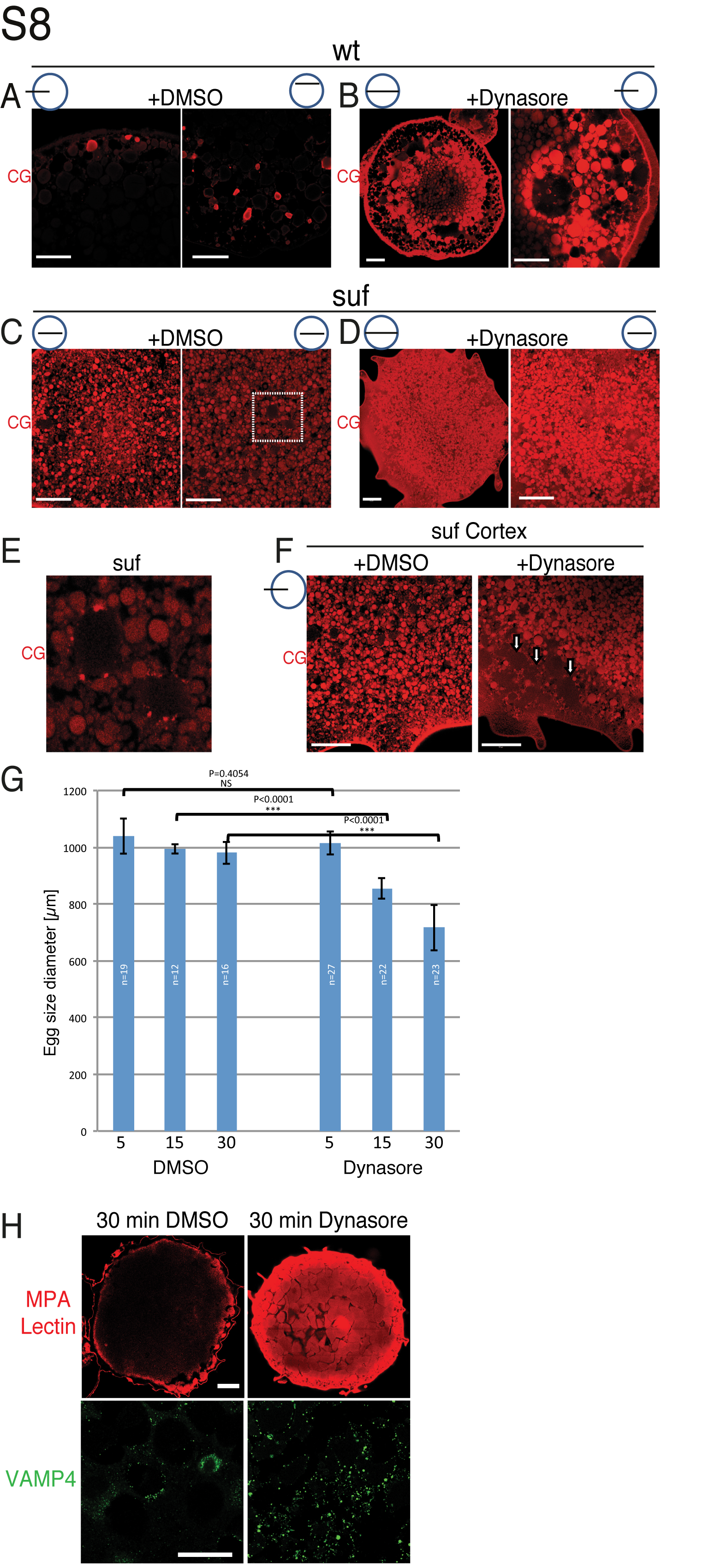

Supplement: Figure S8 — The Dynamin inhibitor Dynasore mimics the suf/spastizin phenotype. Cortical granules stained with MPA-Lectin (red) in wild type (A, B) or suf/spastizin mutants (C, D) after treatment with the carrier DMSO (A, C) or with Dynasore (B, D). The dashed square in panel C indicates the magnification in panel E. (E) In untreated, suf/spastizin mutants MPA-Lectin (red) foci accumulated on the surface of MPA-negative vesicles similar to the cisternae discovered by EM suggesting that MPA-Lectin cargo is sorted, but not pinched off the compartment. (F) Cortex region of suf/spastizin oocyte before and after Dynasore treatment. Remarkably, suf mutants treated with Dynasore showed cortical granules with weak MPA-Lectin background (white arrowheads), which suggested that Dynasore inhibited sorting and removal of the MPA-Lectin cargo into buds. Small icon next to figures indicates the level of the optical section (black line) in the oocyte (blue circle). Scale bar: 50 µm. (G) Quantification of total egg size shown in Figure 7G. Note the significant size reduction of chorion elevation after 15 min. Error bars represent standard deviation. (H) Cellular marker analysis of ovulated stage V eggs. A 30 min treatment with Dynasore of ovulated eggs leads to an accumulation of MPA-lectin (red) and VAMP4 (green) similar to suf mutants. Scale bar: 50 µm. (TIF) [file pgen.1004449.s008.tif]
